# MAVS is energized by Mff which senses mitochondrial metabolism via AMPK for acute antiviral immunity

**DOI:** 10.1038/s41467-020-19287-7

**Published:** 2020-11-11

**Authors:** Yuki Hanada, Naotada Ishihara, Lixiang Wang, Hidenori Otera, Takaya Ishihara, Takumi Koshiba, Katsuyoshi Mihara, Yoshihiro Ogawa, Masatoshi Nomura

**Affiliations:** 1 Department of Biological Sciences, Graduate School of Science, Osaka University, 1-1 Machikaneyama, Toyonaka, 560-0043 Japan; 2 Department of Medicine and Bioregulatory Science, Graduate School of Medical Science, Kyushu University, 3-1-1 Maidashi, Higashi-ku, Fukuoka, 812-8582 Japan; 3 Department of Protein Biochemistry, Institute of Life Science, Kurume University, 67 Asahi-machi, Kurume, 830-0011 Japan; 4 Department of Medical Biochemistry, Kurume University School of Medicine, 67 Asahi-machi, Kurume, 830-0011 Japan; 5 Department of Molecular Biology, Graduate School of Medical Science, Kyushu University, 3-1-1 Maidashi, Higashi-ku, Fukuoka, 812-8582 Japan; 6 Department of Chemistry, Faculty of Science, Fukuoka University, 8-19-1 Nanakuma, Jonan-ku, Fukuoka, 814-0180 Japan; 7 Department of Endocrinology and Metabolism, Kurume University School of Medicine, 67 Asahi-machi, Kurume, 830-0011 Japan

**Keywords:** Mitochondrial proteins, Energy metabolism

## Abstract

Mitochondria are multifunctional organelles that produce energy and are critical for various signaling pathways. Mitochondrial antiviral signaling (MAVS) is a mitochondrial outer membrane protein essential for the anti-RNA viral immune response, which is regulated by mitochondrial dynamics and energetics; however, the molecular link between mitochondrial metabolism and immunity is unclear. Here we show in cultured mammalian cells that MAVS is activated by mitochondrial fission factor (Mff), which senses mitochondrial energy status. Mff mediates the formation of active MAVS clusters on mitochondria, independent of mitochondrial fission and dynamin-related protein 1. Under mitochondrial dysfunction, Mff is phosphorylated by the cellular energy sensor AMP-activated protein kinase (AMPK), leading to the disorganization of MAVS clusters and repression of the acute antiviral response. Mff also contributes to immune tolerance during chronic infection by disrupting the mitochondrial MAVS clusters. Taken together, Mff has a critical function in MAVS-mediated innate immunity, by sensing mitochondrial energy metabolism via AMPK signaling.

## Abstract (web summary)

Mitochondrial antiviral signalling protein (MAVS) is an adaptor of RIG-I like receptor. Here the authors show that mitochondrial fission factor (Mff) is required for clustering of MAVS on the outer mitochondrial membrane during antiviral response.

## Introduction

Mitochondria are multifunctional organelles that have critical roles not only in energy production by oxidative phosphorylation but also in various cellular signaling pathways such as apoptosis, calcium signaling, oxidative stress, and innate immunity^1–3^. Mitochondria dynamically change their morphology by a balance between active fusion and fission in response to intracellular signaling and the surrounding environment^4,5^. In mammals, mitochondrial membrane fusion is regulated by GTPase proteins: two mitofusin isoforms (Mfn1 and Mfn2) on the outer membrane (OM), and optic atrophy type 1 (OPA1) on the inner membrane^5^. Mitochondrial fission is also driven by another GTPase protein, dynamin-related protein 1 (Drp1). Drp1 is localized primarily in the cytosol, is targeted to mitochondrial fission sites on the OM, and mediates mitochondrial fission^5^. Mitochondrial fission has been implicated in various cellular signaling pathways and diseases, such as apoptosis^4^, embryonic development^6,7^, neurodegeneration^6–8^, mitophagy^5^, metabolic disorders^9,10^, and immunity^11^. Several Drp1 receptors have been identified in mammals, such as mitochondrial fission factor (Mff)^12,13^ and mitochondrial dynamic protein of 49 and 51 kDa (MiD49 and MiD51, respectively)^14–16^. Mff is an OM-anchored protein containing a putative Drp1-binding region in its N-terminus^12,13^. Mutations in Mff cause neurodegenerative disease in humans^17–19^, and gene trap disruption of Mff in mice leads to heart failure, infertility, neuromuscular disorders, and growth abnormalities in several tissues^20^; however, the details of the physiological roles of Mff remain unknown.

RNA virus infection induces the innate immune response, concomitant with mitochondrial morphological changes^1^. Mitochondrial antiviral signaling (MAVS) protein, also known as IPS-1/VISA/Cardif, plays a central role in the induction of the antiviral innate immune response on mitochondria^21–24^. A C-terminal transmembrane domain of MAVS for anchoring to the mitochondrial OM is required for its antiviral activity^22,23^. MAVS is an adaptor of retinoic acid-inducible gene-I-like receptors (RLRs) such as RIG-I and MDA5, which are RNA helicases that recognize intracellular viral double-stranded RNA (dsRNA)^1^. MAVS has an N-terminal caspase-recruitment domain (CARD) that interacts with the CARDs of RLRs^21–24^. Once MAVS is activated via its interaction with RLRs, it forms a highly oligomeric complex^25^ and provides a platform for recruiting downstream signal transducers and kinases, leading to the robust induction of type 1 interferon (IFN) proteins and other inflammatory cytokines^1,21–24^. Several reports have shown that mitochondrial dynamics are related to the MAVS-mediated antiviral response^26–29^. Mfn2 interacts with MAVS and inhibits the antiviral response^26^; however, knockout (KO) of both Mfn isoforms severely arrests MAVS signaling^30^. Meanwhile, treatment with respiratory inhibitors, the increased expression of uncoupling protein 2, or mutations in mitochondrial DNA (mtDNA) also lead to the arrest of MAVS signaling^30,31^. However, the molecular mechanism by which mitochondria regulate the antiviral response and the reason why the immune response occurs on mitochondria remain largely unknown.

Mitochondrial dynamics and signaling are linked to energy homeostasis and metabolism^32,33^. Under respiratory dysfunction, the transmembrane domain of long-form OPA1 is processed to generate short-form OPA1, leading to mitochondrial fragmentation via the attenuation of mitochondrial fusion^34^. In contrast, under energy starvation, Drp1 is phosphorylated, resulting in mitochondrial elongation as a mechanism to escape from mitophagy^35,36^. Recent studies have demonstrated that Mff is phosphorylated by AMP-activated protein kinase (AMPK), leading to the activation of mitochondrial fission^37,38^. Immune responses are known to be susceptible to energy status, as suggested by in vivo analysis of mice^39,40^ and cell-based analysis^41^; however, the molecular mechanism for the energetic regulation of innate immune signaling has not been characterized in depth.

Here we show an important function for Mff in the regulation of MAVS. Mff mediates MAVS cluster formation on the mitochondrial OM to activate the innate immune response, in a membrane fission-independent manner. The dual functions of Mff in mitochondrial fission and MAVS activity are both regulated by AMPK in response to energetic conditions. In addition, after acute activation, MAVS clusters are lost from the mitochondria, switching off the antiviral response. Thus Mff switches from a strong and transient immune response under energy-rich conditions to a weak and continuous immune response under energy-starved conditions. Taken together, our results provide evidence for a missing link between bioenergetics and the innate immune response on mitochondria.

## Results

### Mff is required for the MAVS-mediated antiviral response

**Fig. 1:**
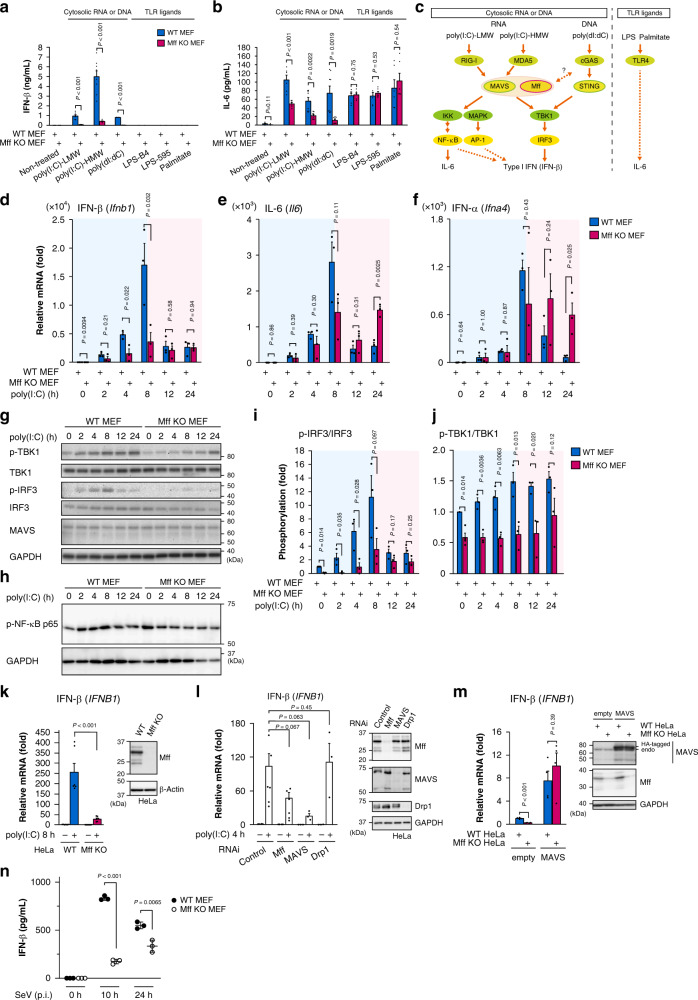
Mff is required for MAVS-mediated antiviral signaling. **a**, **b** IFN-β (**a**) and IL-6 (**b**) secretion in WT and Mff KO MEFs. Cells were transfected with poly(I:C)-LMW (WT, KO: *n* = 6, 5 in **a**; *n* = 6 in **b**), poly(I:C)-HMW (*n* = 8), or poly(dI:dC) (WT, KO: *n* = 4, 5 in **a**; *n* = 8 in **b**), or cultured with LPS or palmitate (*n* = 6) for 8 h. *P* values; two-tailed unpaired *t* test. **c** Scheme of the innate immune cascades examined in this study. Defects of innate immune response were observed around MAVS under Mff deficiency. **d**–**f** qRT-PCR analyses of IFN-β (**d**), IL-6 (**e**), and IFN-α (**f**) mRNA in WT and Mff KO MEFs transfected with poly(I:C)-LMW (1 μg/mL) for the indicated times. *P* values; two-tailed unpaired *t* test (*n* = 3). The blue shaded parts of the graphs (0–8 h) represent the acute phase after poly(I:C), and the red shaded parts (8–24 h) represent the tolerant phase. **g**–**j** Immunoblot analyses of IRF3, TBK1 (**g**) and NF-κB (**h**) phosphorylation as in **d**–**f**. Phosphorylation levels of IRF3 and TBK1 were normalized to its total protein levels. *P* values; two-tailed unpaired *t* test (2–24 h; *n* = 3) or Mann–Whitney’s *U* test (0 h; *n* = 4). **k**, **l** qRT-PCR analysis of IFN-β mRNA in WT and Mff KO HeLa cells (**k**) or in HeLa cells transfected with control, Mff, MAVS, or Drp1 siRNA (**l**). Cells were transfected with poly(I:C)-LMW (1 μg/mL) for the indicated times. The protein levels of Mff and other RNAi genes were analyzed by immunoblotting. *P* values; two-tailed unpaired *t* test (**k**, *n* = 6; **l**, Control, Mff, MAVS, Drp1: *n* = 6, 6, 3, 3). **m** qRT-PCR analysis of IFN-β mRNA in WT HeLa and Mff KO HeLa cells transfected with empty vector or HA-tagged MAVS. *P* values; two-tailed unpaired *t* test (*n* = 4). Overexpressed MAVS protein was detected by immunoblotting. **n** IFN-β secretion in WT and Mff KO MEFs infected with Sendai virus (SeV) at 10 and 24 h post infection (p.i.). *P* values; two-tailed unpaired *t* test (*n* = 3). Data represent means ± SEM from three independent experiments (**a**, **b**, **d**–**f**, **i**, **j**–**m**) or representative one experiment (**n**) with similar results. Immunoblot analyses were repeated independently three times with similar results. Source data are provided as a Source data file.

Mff is a mitochondrial OM protein that mediates the recruitment of the mitochondrial fission-stimulating GTPase Drp1 to mitochondrial fission sites^5^, which is enhanced by AMPK-dependent phosphorylation under respiratory dysfunction^38^. To investigate the roles of Mff, we initially established Mff KO mouse embryonic fibroblasts (MEFs; Supplementary Fig. 1a–d). As expected, Mff KO MEFs had elongated mitochondria (Supplementary Fig. 1c) and failed to recruit Drp1 to the mitochondria (Supplementary Fig. 1d). Next, we examined the role of Mff in MAVS-mediated antiviral innate immunity. Type I IFN family proteins, including several isoforms of IFN-α and the single isoform of IFN-β, are antiviral cytokines that are induced to inhibit viral replication^42^. After introducing the low molecular weight (LMW) form of poly(I:C), a viral dsRNA analog, the secretion of IFN-β and interleukin 6 (IL-6) analyzed by enzyme-linked immunosorbent assay (ELISA) was suppressed severely in Mff KO MEFs (Fig. 1a, b, “poly(I:C)-LMW”), suggesting that Mff plays a critical role in the MAVS-mediated antiviral response. Mitochondrial morphological changes after the viral RNA introduction^27,29,43,44^ were severely repressed in Mff KO MEFs and in Drp1 KO MEFs (Supplementary Fig. 1e). Drp1 recruitment was also severely repressed in Mff KO MEFs (Supplementary Fig. 1f). The immediate fragmentation of mitochondria in wild-type (WT) MEFs was subsequently recovered to normal mitochondrial morphology toward elongation under the antiviral response (Supplementary Fig. 1e), as reported^27^. These data suggested that Mff is required for cytokine induction and Drp1-dependent mitochondrial fission during the antiviral response.

MAVS mediates signal transduction from RNA sensors to kinases and transcription factors, such as TANK-binding kinase 1 (TBK1) and interferon regulatory factor 3 (IRF3), for IFN-β induction^1^. When we introduced various concentrations of poly(I:C)-LMW, the antiviral response occurred in a poly(I:C) concentration-dependent manner, and the response was severely repressed in the absence of Mff (Supplementary Fig. 2a, b). When we tested other pathogens (Fig. 1c), high molecular weight (HMW) form of poly(I:C)-induced cytokine secretion was also suppressed in Mff KO cells (Fig. 1a, b). Unexpectedly, the introduction of poly(dI:dC), an analog of cytosolic viral dsDNA, also induced cytokine secretion in an Mff-dependent manner (Fig. 1a, b), although the intracellular DNA only weakly induced transcription of these cytokines (Supplementary Fig. 2a, b). It is possible that the MAVS pathway could be activated by cross-talk with the cGAS/STING pathway, known to be activated by viral DNAs (Fig. 1c), as previously reported^27,45,46^, although the molecular details remain to be determined. In contrast, the production of IL-6 via Toll-like receptor 4 (TLR4), which is independent of MAVS^24^ and can be stimulated by TLR4 ligands such as lipopolysaccharide (LPS) and palmitate^47^, was comparable between WT and Mff KO MEFs (Fig. 1b). These results suggested that Mff specifically modulates the MAVS-mediated antiviral response (Fig. 1c).

Next we analyzed the time profile of the MAVS-mediated antiviral response. When we examined the mRNA expressions of antiviral cytokines in WT MEFs, IFN-β (Fig. 1d), IL-6 (Fig. 1e), and IFN-α (Fig. 1f) were induced until 8 h after poly(I:C) transfection (acute phase), and their expression was drastically suppressed after 8 h (tolerance phase). A similar temporal response was observed for the level of phosphorylation of IRF3 in WT MEFs analyzed by immunoblotting (Fig. 1g, i). In contrast, the acute antiviral response for cytokine mRNA induction (Fig. 1d–f) and IRF3 phosphorylation (Fig. 1g, i) was suppressed in Mff KO MEFs. The phosphorylation of TBK1 (Fig. 1g, j) and activation of nuclear factor-κB (Fig. 1h, “p-NF-κB p65”) modestly induced in WT MEFs was also repressed in Mff KO MEFs. These results suggest that Mff regulates signal transduction of the MAVS-mediated antiviral response.

To examine the involvement of Mff in the antiviral response in human cells, we analyzed HeLa cells and found the impaired induction of IFN-β mRNA in Mff KO HeLa cells^16^ (Fig. 1k) and in Mff- or MAVS-repressed HeLa cells by RNA interference (RNAi; Fig. 1l). In contrast, the antiviral cytokine IFN-β was induced normally in Drp1-RNAi HeLa cells, as in control cells (Fig. 1l), suggesting that mitochondrial fission is not essential for the antiviral response, consistent with previous reports of Drp1 depletion or the expression of a dominant-negative mutant Drp1 K38A^27,48^. It was also reported that the MAVS overexpression causes the induction of IFN proteins without virus infection, possibly by auto-activation^21–24^. When MAVS was overexpressed in Mff KO HeLa cells, IFN-β was highly induced, as observed in WT HeLa cells (Fig. 1m), suggesting that Mff acts upstream of MAVS activation and the subsequent induction of type I IFN. Taken together, we found that Mff selectively affects the MAVS-mediated antiviral signaling pathway.

We further examined the function of Mff during RNA virus infection. At 10 h after Sendai virus (SeV) infection, IFN-β secretion observed in WT MEFs was severely repressed in Mff KO MEFs (Fig. 1n). However, the antiviral response in Mff KO cells was prolonged for 24 h after RNA virus infection, although the antiviral response was exhausted in WT cells (Fig. 1n). These results confirmed that Mff regulates MAVS-mediated anti-RNA viral response in a time-dependent manner.

### Mff mediates MAVS cluster formation on mitochondria

**Fig. 2:**
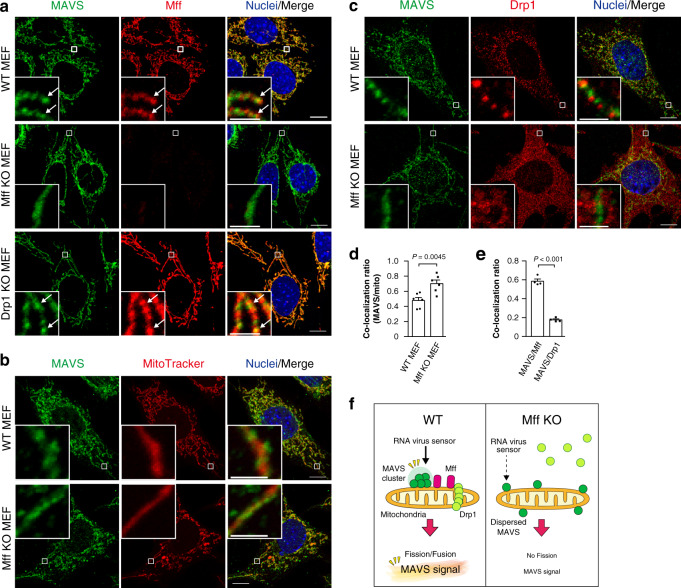
Mff regulates accumulated distribution of MAVS on mitochondria. **a** Confocal microscopic images of MAVS and Mff. WT, Mff KO, and Drp1 KO MEFs were immunostained with anti-MAVS (green) and anti-Mff (red) antibodies. **b** Confocal microscopic images of MAVS and mitochondria. WT and Mff KO MEFs were stained with MitoTracker Red (mitochondria; red) and immunostained with an anti-MAVS antibody (green). **c** Confocal microscopic images of MAVS and Drp1. WT and Mff KO MEFs were immunostained with anti-MAVS (green) and anti-Drp1 (red) antibodies. Nuclei were stained with Hoechst 33342 (blue) in **a–c**. Scale bars: 10 μm (whole) and 2 μm (magnified) in **a–c**. **d** Co-localization ratio between MAVS and mitochondrial tubules (mitoRFP) in WT and Mff KO MEFs. *P* value; two-tailed unpaired *t* test (*n* = 6). **e** Co-localization ratio between MAVS and Mff or Drp1 in WT MEFs. *P* value; two-tailed unpaired *t* test (*n* = 4). **f** Working hypothesis for the accumulated distribution of MAVS and the mitochondrial antiviral response regulated by Mff. Data represent means ± SEM examined over more than three biologically independent samples (**d**, **e**). All microscopic analyses were repeated independently more than three times with similar results. Source data are provided as a Source data file.

Under antiviral signaling, MAVS forms huge oligomeric complexes on mitochondria^25^. When we visualized endogenously expressed MAVS in MEFs by immunofluorescence confocal microscopy, MAVS formed many accumulated dot-like clusters on mitochondrial tubules (Fig. 2a–c), consistent with previous observations under super-resolution microscopy^43^. To examine the role of Mff in the distribution of MAVS, we analyzed Mff KO MEFs and found a more diffuse distribution of MAVS on mitochondrial tubules (Fig. 2a, b), as well as the cytoplasmic distribution of Drp1 (Fig. 2c and Supplementary Fig. 1d). Then we measured the co-localization efficiency between MAVS and mitochondria. Because dispersal of MAVS clusters causes MAVS to have a more uniform distribution on mitochondria, this can be measured as an increase in the correlational coefficient between MAVS and mitochondrial signals. The quantification confirmed that Mff mediates the formation of MAVS clusters, which accumulated on a part of the mitochondria (Fig. 2d). Under double staining of MAVS and Mff, Mff partly but clearly overlapped with the MAVS clusters in WT MEFs and Drp1 KO MEFs (Fig. 2a, quantification in Fig. 2e). In contrast, the MAVS clusters rarely colocalized with Drp1 structures in WT MEFs (Fig. 2c, quantification in Fig. 2e). We further analyzed oligomerization of MAVS biochemically using clear-native polyacrylamide gel electrophoresis (CN-PAGE), showing that the formation of higher-order MAVS oligomers was repressed in Mff KO cells (Supplementary Fig. 2c). These data suggested that Mff regulates the mitochondrial distribution of MAVS, independently of Drp1-dependent mitochondrial fission (model: Fig. 2f).

### Mff regulates antiviral response independent of fission

**Fig. 3:**
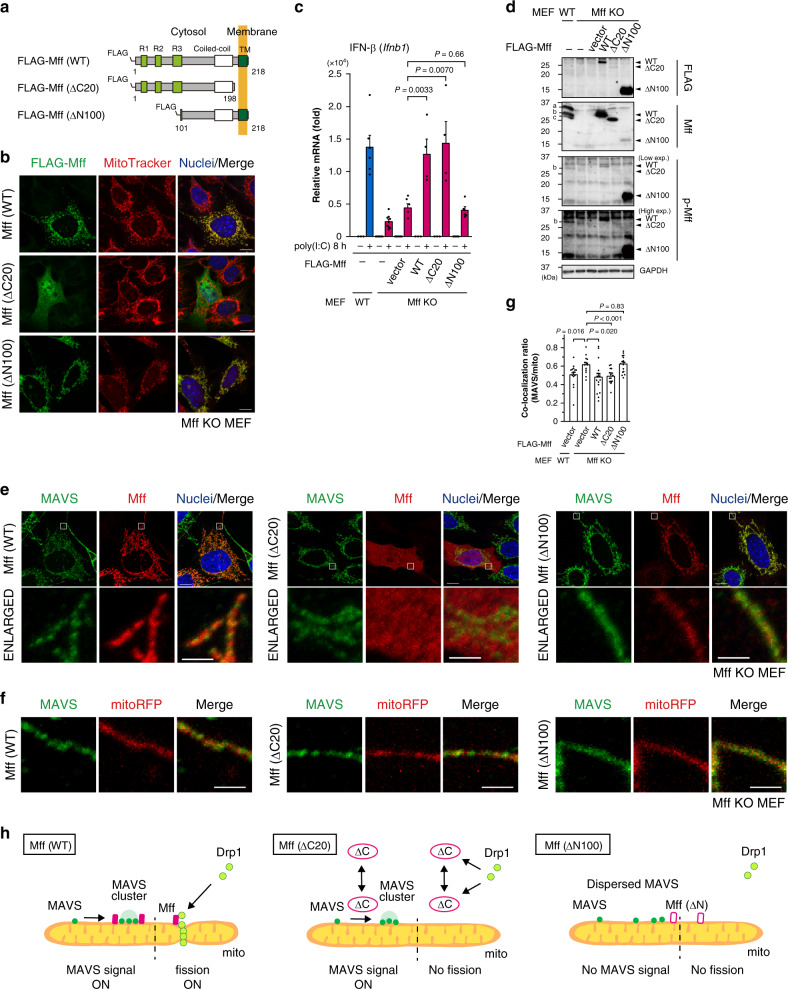
Mff regulates MAVS-mediated antiviral response independent of mitochondrial fission. **a** Scheme of murine Mff deletion constructs. The amino acid numbering scheme used in the constructs corresponds to splice isoform 8 of Mff^13^. TM: transmembrane domain. **b** Confocal microscopic images of Mff and mitochondria. Mff KO MEFs were transfected with expression vectors for FLAG-tagged Mff (WT), Mff (ΔC20), or Mff (ΔN100), stained with MitoTracker Red (mitochondria; red), and immunostained with an anti-FLAG antibody (green). Nuclei were stained with DAPI (blue). Scale bars: 10 μm. **c** qRT-PCR analysis of IFN-β mRNA in WT MEFs, Mff KO MEFs, and Mff KO MEFs transfected with an empty vector or expression vectors for FLAG-tagged Mff (WT), Mff (ΔC20), or Mff (ΔN100). Cells were transfected with or without poly(I:C)-LMW (1 μg/mL) for 8 h. Data represent means ± SEM from three independent experiments (vector, WT, ΔC20, ΔN100: *n* = 6, 4, 4, 5, *P* value; two-tailed unpaired *t* test). **d** Immunoblot analyses of endogenous and exogenous Mff and Mff phosphorylation in the cells listed in **c**. The indicated letters (a, b, c) correspond to the endogenous Mff bands observed in WT MEFs. **e** Confocal microscopic images of MAVS and Mff. Mff KO MEFs transfected with expression vectors for Mff (WT), Mff (ΔC20), or Mff (ΔN100) were immunostained with anti-MAVS (green) and anti-Mff (red) antibodies. Nuclei were stained with Hoechst 33342 (blue). A magnified view of the boxed region is shown below. Scale bars: 10 μm (whole) and 2 μm (magnified). **f** Magnified images of MAVS on mitochondria in Mff KO MEFs co-transfected with each Mff mutant and a mitochondrial marker mitoRFP (red). Cells were immunostained with anti-MAVS antibody (green). Scale bars: 2 μm. **g** Co-localization ratio between MAVS and mitoRFP in WT MEFs or Mff KO MEFs co-transfected with mitoRFP and an empty vector or each Mff mutant. Data represent means ± SEM (WT-vector, Mff KO-vector, Mff KO-WT, Mff KO-ΔC20, Mff KO-ΔN100: *n* = 15, 13, 16, 16, 16, *P* value; two-tailed unpaired *t* test). **h** Working hypothesis for the coupling of the distribution of MAVS and mitochondrial fission by Mff. mito: mitochondria. Immunoblot and microscopic analyses were repeated independently three times with similar results. Source data are provided as a Source data file.

As shown above, MAVS failed to form clusters and failed to mediate an efficient antiviral response in Mff-depleted cells. To further analyze the underlying molecular mechanism, we examined the domain structure of Mff protein required for the MAVS-mediated antiviral response. We introduced three different FLAG-tagged Mff constructs into Mff KO MEFs (Fig. 3a): full-length Mff (“Mff [WT]”); Mff lacking the C-terminal transmembrane domain (“Mff [ΔC20]”), and Mff lacking 100 amino acid residues from the cytoplasmic N-terminal domain (“Mff [ΔN100]”)^13^. After transient expression of these constructs in Mff KO MEFs, their distribution (Fig. 3b), immune response (Fig. 3c), and protein expression (Fig. 3d) were analyzed.

In Mff KO MEFs, Mff (WT) and Mff (ΔN100) localized to the mitochondria, while Mff (ΔC20) was localized in the cytoplasm (Fig. 3b), because it did not have the transmembrane domain necessary for anchoring to the OM^13^. The highly elongated mitochondria in Mff KO MEFs were changed to fragmented mitochondria by Mff (WT) expression but not by Mff (ΔC20) or Mff (ΔN100) expression (Fig. 3b and Supplementary Fig. 3), and these two truncated mutants were unable to recruit Drp1 to the mitochondria (Supplementary Fig. 3a), as reported previously^13^. As expected, the antiviral response (Fig. 3c) and MAVS cluster formation (Fig. 3e, f) were rescued by Mff (WT) expression in Mff KO MEFs. In contrast, although Mff (ΔN100) was highly expressed (shown in the upper panel of Fig. 3d, immunoblot detected by an anti-FLAG antibody), Mff (ΔN100) expression failed to rescue the diffuse mitochondrial distribution of MAVS (Fig. 3e, f) and the defective antiviral signaling (Fig. 3c) in Mff KO MEFs, suggesting that the N-terminal region of Mff is critical for mitochondrial fission and antiviral signaling. Next we analyzed Mff (ΔC20); the protein expression level was lower than that of Mff (WT) (Fig. 3d, detected by anti-FLAG). Unexpectedly, although Mff (ΔC20) lost the Drp1-dependent mitochondrial fission activity (Fig. 3b, e and Supplementary Fig. 3), Mff (ΔC20) expression rescued the antiviral response (Fig. 3c) and MAVS cluster formation on mitochondria (Fig. 3e, f). Quantification of MAVS cluster formation on mitochondria as Fig. 2d further confirmed that Mff (ΔC20) significantly rescued MAVS cluster formation (Fig. 3g). In conclusion, Mff has dual functions: it promotes mitochondrial fission via Drp1 recruitment to the mitochondria, and it mediates the antiviral response via MAVS cluster formation, which is independent of mitochondrial fission (model: Fig. 3h).

### Mff phosphorylation by AMPK results in defective antiviral response

**Fig. 4:**
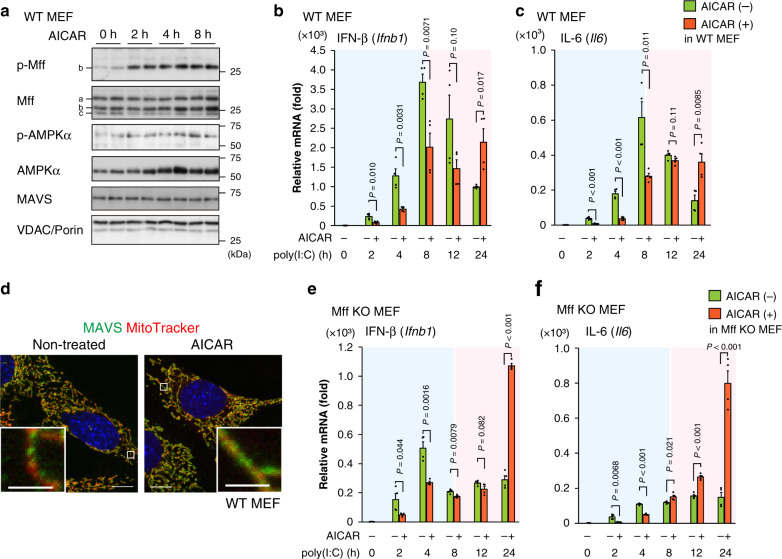
The acute antiviral response is decreased in AICAR-treated cells with Mff phosphorylation. **a** WT MEFs were treated with the AMPK activator AICAR (2 mM) for the indicated times. Whole-cell lysates were analyzed by immunoblotting using antibodies against the indicated proteins. The indicated letters (a, b, c) correspond to the endogenous Mff bands observed in WT MEFs. **b**, **c** qRT-PCR analyses of IFN-β (**b**) and IL-6 (**c**) mRNA in WT MEFs transfected with poly(I:C)-LMW (1 μg/mL) in the absence (−) or presence (+) of AICAR (2 mM, pretreated for 2 h) for the indicated times. **d** WT MEFs were stained with MitoTracker Deep Red (mitochondria; red) and then treated with or without AICAR (2 mM) for 8 h. After fixation and permeabilization, cells were immunostained with an anti-MAVS antibody (green). Nuclei were stained with Hoechst 33342 (blue). Scale bars: 10 μm (whole) and 2 μm (inset). **e**, **f** qRT-PCR analyses of IFN-β (**e**) and IL-6 (**f**) mRNA in Mff KO MEFs transfected with poly(I:C)-LMW (1 μg/mL) in the absence (−) or presence (+) of AICAR (2 mM, pretreated for 2 h) for the indicated times. The blue shaded parts of the graphs (0–8 h) represent the acute phase after poly(I:C), and the red shaded parts (8–24 h) represent the tolerant phase in **b**, **c**, **e**, **f**. Data represent means ± SEM from two independent experiments (*n* = 4, *P* values; two-tailed unpaired *t* test) in **b**, **c**, **e**, **f**. Immunoblot analyses and all microscopic analyses were repeated independently more than three times with similar results. Source data are provided as a Source data file.

**Fig. 5:**
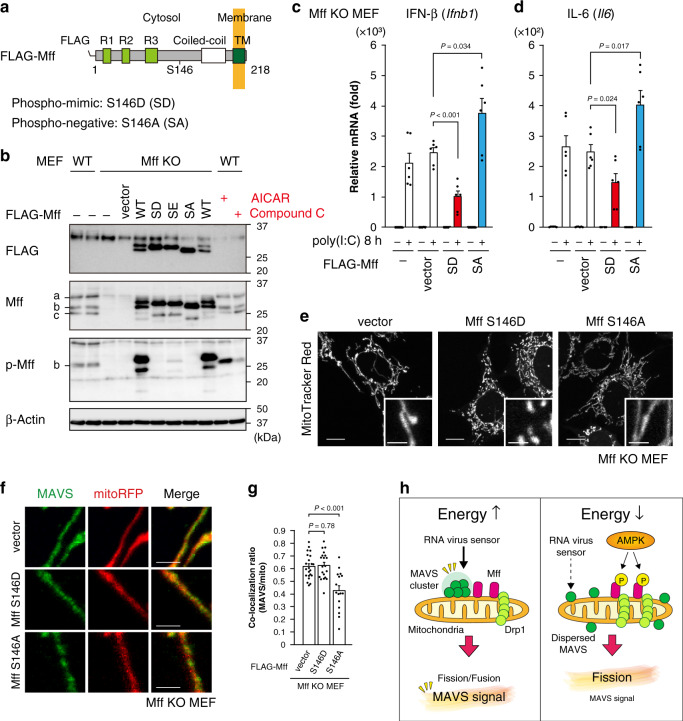
Mff phosphorylation impairs mitochondrial antiviral response. **a** Scheme of the murine phosphorylation-negative/mimic Mff constructs used in this study. A point mutation was introduced into FLAG-tagged Mff (WT) at S146 as indicated. **b** Mff KO MEFs stably expressing each Mff construct were analyzed by immunoblotting using anti-FLAG, anti-Mff, and anti-phospho-Mff antibodies. SD: S146D, SE: S146E, SA: S146A. WT MEFs treated with AICAR (AMPK activator: 2 mM) or compound C (AMPK inhibitor: 20 μM) for 2 h were used as controls. **c**, **d** qRT-PCR of IFN-β (**c**) and IL-6 (**d**) mRNA in Mff KO MEFs stably expressing each Mff construct with or without poly(I:C)-LMW (1 μg/mL) transfection for 8 h. Data represent means ± SEM of three independent experiments (*n* = 6, *P* values; two-tailed unpaired *t* test). **e** Representative live-cell images of MitoTracker Red (mitochondria, white) in Mff KO MEFs stably expressing each Mff construct. Scale bars: 10 μm (whole) and 2 μm (inset). **f** Magnified images of MAVS on mitochondria in Mff KO MEFs stably expressing each Mff construct. The cells were transiently transfected with a mitochondrial marker mitoRFP (red) and immunostained with an anti-MAVS antibody (green). Scale bars: 2 μm. **g** Co-localization ratio between MAVS and mitoRFP in Mff KO MEFs stably expressing each Mff construct. Data represent means ± SEM (vector, SD, SA: *n* = 22, 20, 16, *P* values; two-tailed unpaired *t* test). **h** Working hypothesis for the energetic regulation of the mitochondrial antiviral response by Mff phosphorylation. Immunoblot analyses and all microscopic analyses were repeated independently more than three times with similar results. Source data are provided as a Source data file.

Mff is phosphorylated by AMPK under nutrient-starved conditions^38^; however, its physiological meaning is not understood. Here we examined the role of AMPK in the MAVS-mediated antiviral response. Consistent with previous studies^37,38^, treatment with the AMPK activator AICAR increased the level of Mff phosphorylation, as well as the AMPKα phosphorylation (Fig. 4a). In the presence of AICAR, the induction of antiviral cytokines (IFN-β and IL-6) was clearly repressed at the acute phase (before 8 h), while the mild antiviral response was still maintained at the tolerant phase (after 8 h) (Fig. 4b, c), suggesting that the acute and temporal antiviral response is suppressed but prolonged by AMPK. In the absence of AICAR, phosphorylation of AMPKα and Mff was not significantly changed during the antiviral response in WT cells, suggesting that the poly(I:C) does not directly induce AMPK activation (Supplementary Fig. 2d). The mild and prolonged antiviral response observed after AICAR treatment (Fig. 4b, c) was similar to that seen in Mff KO cells (Fig. 1d, e). Under fluorescent microscopy, the accumulated mitochondrial distribution of MAVS became more diffuse after AICAR treatment (Fig. 4d), further suggesting that AMPK is involved in the distribution and activation of MAVS on mitochondria. We further analyzed the antiviral response in Mff KO MEFs and found that AICAR treatment until 8 h only weakly affected this response (Fig. 4e, f), suggesting that the inhibitory effect of AICAR on acute cytokine induction largely requires Mff. To examine further the role of Mff phosphorylation by AMPK, we constructed Mff proteins that were mutated at the S146 phosphorylation site and stably expressed them in Mff KO MEFs (Fig. 5a, b). We found that the phosphorylation-mimic mutant S146D migrated slower than the phosphorylation-negative mutant S146A in sodium dodecyl sulfate (SDS)-PAGE, similar to phosphorylated and non-phosphorylated Mff (WT), respectively (Fig. 5b). Under the infectious condition, S146A rescued cytokine induction in Mff KO MEFs (Fig. 5c, d), without inducing apparent mitochondrial fission (Fig. 5e), but it induced MAVS cluster formation (images in Fig. 5f, quantification in Fig. 5g). In contrast, S146D rather inhibited cytokine induction (Fig. 5c, d), which induced mitochondrial fission (Fig. 5e), without inducing MAVS cluster formation (Fig. 5f, g). Unexpectedly, stably expressed Mff (WT) failed to activate the cytokine induction (Supplementary Fig. 4e, f), different from those of transiently expressed Mff (WT) that rescued antiviral response in Mff KO cells (Fig. 3c). Stably expressed Mff (WT) was excessively phosphorylated at S146 (Fig. 5b), which should cause impaired antiviral response, although the molecular details remain unknown. Thus Mff phosphorylation by AMPK repressed the antiviral response, concomitant with the activation of mitochondrial fission (model: Fig. 5h).

### Mitochondrial metabolism controls MAVS via Mff phosphorylation

MAVS signaling is repressed in various conditions affecting mitochondria such as the depletion of mitochondrial fusion factors^30^, treatment with respiratory inhibitors^30,31^, or in cells with mutated mtDNA^31^. In addition, RNAi of mitochondrial fusion proteins^49^, but not mitochondrial fission proteins^50^, causes respiratory dysfunction in various types of cultured cells. However, the molecular mechanism by which mitochondrial dynamics and activity modulate the antiviral response is less well understood. Here we examined the role of the AMPK–Mff axis in the induction of the antiviral response under various mitochondrial respiration-stressed conditions.

**Fig. 6:**
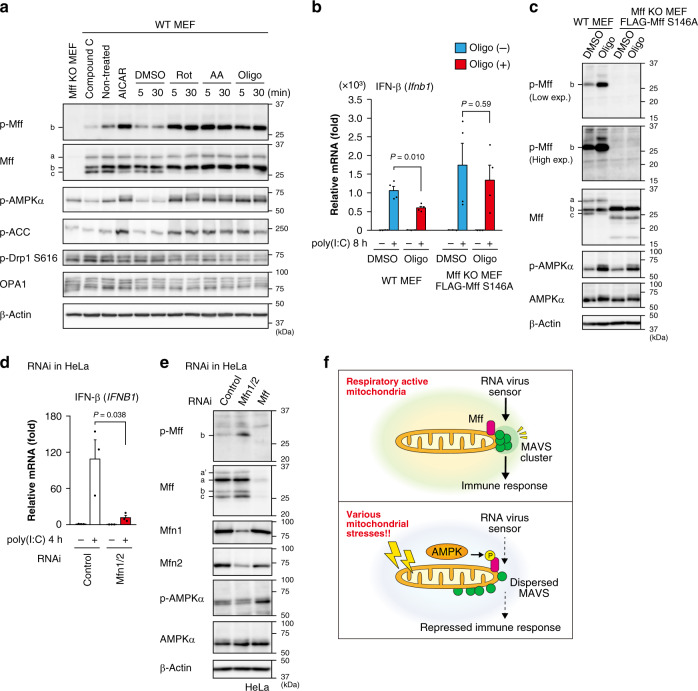
Mitochondrial dysfunction leads to Mff phosphorylation and impaired antiviral response. **a** WT MEFs were treated with the respiratory inhibitors rotenone (Rot, 0.5 μM) or antimycin A (AA, 1 μM) or the ATP synthase inhibitor oligomycin (Oligo, 1 μM) for 5 or 30 min. Whole-cell lysates were analyzed by immunoblotting using antibodies against the indicated proteins. AICAR (2 mM) or compound C (20 μM) was used as an AMPK activator or inhibitor, respectively. DMSO: dimethyl sulfoxide. **b** qRT-PCR analysis of IFN-β mRNA with or without poly(I:C)-LMW in WT MEFs and Mff KO MEFs stably expressing FLAG-Mff S146A. Cells were pretreated with or without oligomycin (Oligo, 1 μM) for 30 min and then transfected with poly(I:C)-LMW (1 μg/mL) in the absence (−) or presence (+) of oligomycin for 8 h. Data represent means ± SEM of two independent experiments (*n* = 4, *P* values; two-tailed unpaired *t* test). **c** Immunoblot analyses of Mff and Mff phosphorylation in WT MEFs and Mff KO MEFs stably expressing FLAG-Mff S146A with or without oligomycin (1 μM, 30 min). The indicated letters (a, b, c) correspond to the endogenous Mff bands observed in WT MEFs. **d** HeLa cells were transfected with control or Mfn1/2 siRNA and cultured for 72 h. The cells were transfected with or without poly(I:C)-LMW (1 μg/mL) for 4 h, and IFN-β induction was analyzed by qRT-PCR. Data represent means ± SEM of three independent experiments (*n* = 3, *P* values; two-tailed unpaired *t* test). **e** Analysis of Mff phosphorylation by immunoblotting using anti-phospho-Mff and anti-Mff antibodies in HeLa cells with the indicated RNAi. The efficiency of RNAi and AMPK activation were also examined by each antibody. **f** Working hypothesis for how mitochondrial dysfunction leads to the suppression of the antiviral response by the AMPK–Mff axis. Immunoblot analyses were repeated independently three times with similar results. Source data are provided as a Source data file.

Treatment with respiratory inhibitors induced the phosphorylation of Mff by AMPK (Fig. 6a) as well as acetyl-CoA carboxylase (ACC) phosphorylation, as reported previously^38^. As expected, treatment with oligomycin, an inhibitor of mitochondrial ATP synthase, induced Mff phosphorylation (Fig. 6a) and repressed the induction of IFN-β (Fig. 6b), suggesting that the phosphorylation of Mff by AMPK is activated by reduced ATP production under mitochondrial dysfunction, thereby impairing the antiviral response. We also confirmed that the protein band shift (from “c” to “b” in Fig. 6a) induced by AICAR and respiratory inhibitors corresponded with Mff phosphorylation. In Mff KO MEFs expressing the phosphorylation-negative mutant S146A, oligomycin treatment had a reduced impact on the antiviral response (Fig. 6b), even when AMPK was activated (Fig. 6c), confirming that the AMPK-dependent phosphorylation of Mff repressed the antiviral response under mitochondrial dysfunction (Fig. 6f).

Next, we examined the role of mitochondrial dynamics by fusion and fission in the antiviral response. As shown above, Drp1 RNAi caused mitochondrial elongation (Supplementary Fig. 4j) but had no effect on the antiviral response (Fig. 1l), consistent with the notion that it only weakly affects respiratory activity^50^. We further found that the antiviral response in Drp1-depleted cells was significantly repressed by AICAR treatment as in control cells (Supplementary Fig. 4a–d), while mitochondria keep highly elongated structures (Supplementary Fig. 4a), suggesting again that AMPK–Mff should have important role in the antiviral response independent of mitochondrial fission. It is noteworthy that the phosphorylation levels of AMPKα and the AMPK substrate ACC in WT MEFs were comparable with those of Mff KO MEFs (Fig. 6a), suggesting that Mff repression should not have a drastic impact on mitochondrial energetics. In contrast, double RNAi of Mfn1 and Mfn2, which has been reported to affect respiration^49,51^, led to an efficient Mff phosphorylation (Fig. 6e) and a defective antiviral response (Fig. 6d) as reported^30^. Under Mfn1/2 depletion, the p-AMPKα band migrated more slowly, possibly due to phosphorylation, compared with control or Mff-depleted cells (Fig. 6e), also confirming that MAVS signaling should be regulated under AMPK. These data suggested that the repressed antiviral signaling in Mfn1/Mfn2-deficient cells should be caused by the AMPK-dependent Mff phosphorylation. We further investigated how mitochondria affect Mff phosphorylation by AMPK. In contrast to stably expressed Mff (Fig. 5b), transiently expressed Mff (WT) was only partly phosphorylated at S146 (Fig. 3d) and activated the antiviral response (Fig. 3c). In the same condition, Mff (ΔN100), which localized to mitochondria, was efficiently phosphorylated on mitochondria, although Mff (ΔC20), which localized to the cytoplasm, was only rarely phosphorylated (Fig. 3d). These data are well consistent with the efficient antiviral activity of Mff (ΔC20) (Fig. 3c), even when localized in the cytoplasm. From these data, we concluded that the mitochondrial localization of Mff is important for the AMPK-dependent regulation. Taken together, the MAVS-mediated antiviral response is suppressed under various respiration-stressed conditions via Mff phosphorylation by AMPK, possibly sensing the production of ATP by mitochondria (model: Fig. 6f).

### Mff inactivates MAVS at the late phase of antiviral response

**Fig. 7:**
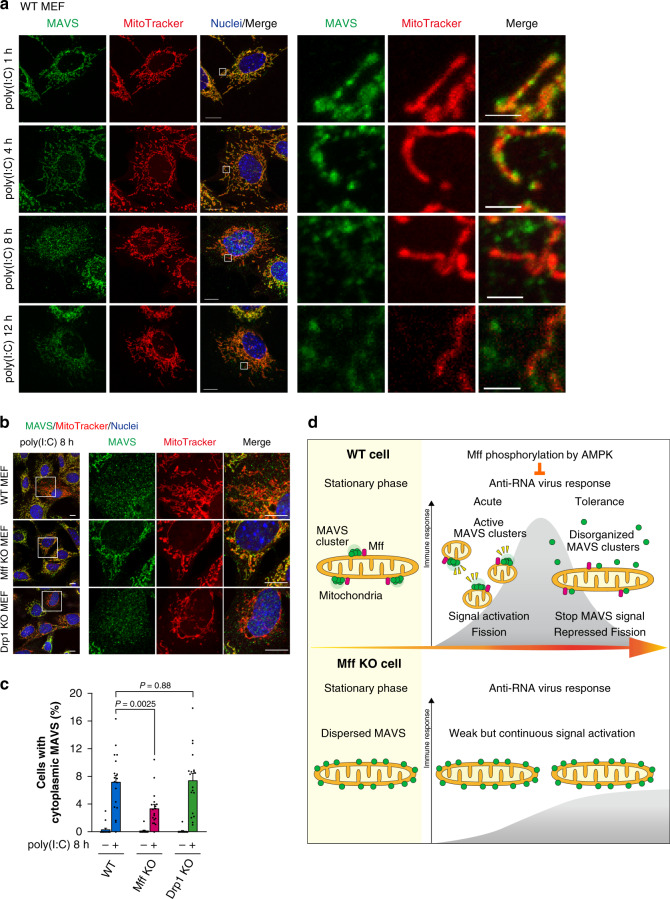
Clearance of mitochondrial MAVS clusters by Mff. **a** Confocal microscopic images of MAVS and mitochondria. WT MEFs were transfected with poly(I:C)-LMW (1 μg/mL) for the indicated times. Cells were stained with MitoTracker Red (mitochondria; red) and immunostained with an anti-MAVS antibody (green). Nuclei were stained with Hoechst 33342 (blue). A magnified view of the boxed region is shown in the right panel. Scale bars: 10 μm (whole) and 2 μm (magnified). **b** WT, Mff KO, and Drp1 KO MEFs were stained with MitoTracker Red (mitochondria; red) and immunostained with an anti-MAVS antibody (green) with poly(I:C)-LMW (1 μg/mL) transfection for 8 h. Nuclei were stained with DAPI (blue). A magnified view of the boxed region is shown in the right panel. Scale bars: 10 μm. **c** The percentage of cells with cytoplasmic MAVS (MAVS signal without MitoTracker Red) was calculated. Data represent means ± SEM of three independent experiments (WT, Mff KO, Drp1 KO; *n* = 18, 18, 19, *P* values; two-tailed unpaired *t* test). **d** Working hypothesis for the mitochondrial membrane subdomains with MAVS clusters in antiviral innate immunity, dynamically regulated by the mitochondrial fission factor Mff and AMPK. All microscopic analyses were repeated independently more than three times with similar results. Source data are provided as a Source data file.

Our data suggested that the induction and termination of the temporal innate immune response are controlled by Mff (Fig. 1d–f). To investigate the mechanism underlying the repression of the MAVS-mediated innate immune response at the tolerant phase, we examined time course of the MAVS distribution during the antiviral response (Fig. 7a, b). The MAVS clusters observed before the poly(I:C) introduction (Fig. 2a–c) were maintained for 1 and 4 h after poly(I:C) introduction (Fig. 7a). In the early phase of the antiviral response in WT MEFs, many MAVS clusters were observed on mitochondria (Fig. 7a). In contrast, by further prolonged stimulation (after ≥8 h), the MAVS clusters were lost from the mitochondrial surface (Fig. 7a, b), although no AMPK activation was observed at the late stage (after 8 h) (Supplementary Fig. 2d). At the late stage, many MAVS signals were observed in the cytoplasm (Fig. 7a). After prolonged antiviral signaling, the cytoplasmic MAVS signals were not observed in Mff KO MEFs but were observed in Drp1 KO MEFs (Fig. 7b), suggesting that Mff mediates the disorganization of the mitochondrial MAVS clusters independently of mitochondrial fission. Cell count analysis also confirmed our conclusion that Mff, but not Drp1, regulates the formation of the cytoplasmic MAVS (Fig. 7c). Collectively, Mff plays important roles in the distribution of MAVS, which should mediate the acute and temporal antiviral response.

## Discussion

Mitochondria are derived from the endosymbiosis of bacteria; however, they also act as a platform for innate immune signaling through MAVS^21–24^. This antiviral platform is not static because MAVS changes its oligomeric status^25^, and mitochondrial membranes are considered to be dynamic as they undergo frequent cycles of fusion and fission^5^. To investigate the dynamic features of the mitochondrial innate immune signaling pathway, we focused on Mff, which is located on the mitochondrial OM and regulates mitochondrial membrane dynamics under cellular signaling^13,38^. Here we found a role for Mff that was independent of mitochondrial fission and mediates the anti-RNA virus response with MAVS.

RNA virus infection induces mitochondrial morphological changes of elongation^27^ or fission^44^. Mfn2 directly interacts with and inhibits MAVS^26^; however, it is unknown how mitochondrial fusion and fission affect the MAVS-mediated innate immune response. From careful morphological and biochemical analyses, we found that mitochondrial morphology changed over time after the introduction of RNA, from fragmentation to elongation. However, we also showed that mitochondrial fission does not directly affect the antiviral response because antiviral cytokines were induced under mitochondrial fission-deficient conditions (Figs. 1l, 3, and 5); instead, Mff plays a critical role in the mitochondrial distribution of MAVS (Fig. 2). Mff and MAVS are both anchored to the OM via their C-terminal membrane anchor domains. The cytoplasmic domain of Mff recruits Drp1 to mitochondrial fission sites via a transient interaction, which mediates the formation of Drp1 oligomers to constrict and divide the mitochondrial membrane^5^. In this analogy, we speculated that Mff should transiently associate with MAVS on the OM and affect its conformation and/or oligomeric status to form activated MAVS clusters, as we showed in CN-PAGE analysis that Mff regulates the oligomeric status of MAVS (Supplementary Fig. 2c). From biochemical analysis, MAVS forms prion-like oligomeric aggregates that are active on mitochondria^25,52^; even the size of MAVS oligomers is not large in vivo^43^ compared with an estimation from an in vitro study^25^. Here we found that MAVS forms dot-like clusters (Fig. 2), as reported previously^43^. These clusters should be a type of membrane subdomain on the mitochondrial surface for the activation of MAVS. Furthermore, under chronic virus infection, Mff mediates the disorganization of mitochondrial MAVS clusters (Fig. 7), which should lead to immune tolerance. In some cases, an acute and drastic immune response efficiently protects cells from virus infection; however, an excessive and prolonged response could damage host cells^42^. A previous report indicated that apoptosis-related caspases cleave MAVS during the antiviral response^53^, but we failed to find degradation products of MAVS (see Fig. 1g); instead, we found changes in the distribution of MAVS at the signal-termination stage (Fig. 7), although Mff phosphorylation was not significantly enhanced (Supplementary Fig. 2d). Further detailed analysis is needed to determine how MAVS forms clusters and detaches from the mitochondrial membrane mediated by Mff. The dynamic movement of MAVS clusters represents a good target for evaluating the immune response in vivo and for manipulating the antiviral response.

Here we found that the antiviral response is repressed under respiratory dysfunction via Mff phosphorylation by AMPK (Figs. 4–6). It has been reported that the MAVS-dependent antiviral response is repressed by various mitochondrial changes, such as mitochondrial fusion deficiency, treatment with an uncoupler, and mtDNA mutations^30,31^. Under these conditions, mitochondrial respiratory activity should be repressed; thus the reduced supply of ATP from mitochondria enhances AMPK activity around mitochondria, which leads to efficient Mff phosphorylation and repression of the antiviral response. Thus Mff links mitochondrial energy production to the innate immune response on mitochondria. These findings provide a clue to solve why the immune response is activated on mitochondria. Mff is one of the main substrates of AMPK in a part of the energy sensor/regulation system^38^, although the physiological function of this energy-dependent modification is less well understood. It seems reasonable to assume that, under pathogenic as well as energy-limiting conditions, cells manage to survive by preserving energy to suppress drastic cytokine synthesis and by promoting energy production to activate mitochondrial fission. Mitochondria are the location of ATP production; thus it seems appropriate that the ATP-sensing system is located on the mitochondrial surface because a proportion of AMPK is localized on mitochondria^54^. Our findings should provide a missing link beyond metabolism to mitochondrial signaling and immunity and point to the possibility that Mff is a therapeutic target for energy-limiting pathology. However, we need further analysis of the molecular mechanism by which Mff regulates the distribution and function of MAVS and how and why Mff regulates antiviral signaling in response to energy status. It was also reported that repression of Mfn2 alone increases the MAVS-mediated antiviral response^26^, possibly by the direct suppression of MAVS by Mfn2, which appears to be a different mechanism to that reported here, indicating that further detailed analysis is required. Our report reveals an important aspect of the relationship between innate immunity and metabolism, in which mitochondrial dynamics is the key regulator of multifunctional mitochondria.

## Methods

### Cell culture and transfection

MEFs and HeLa cells were cultured in Dulbecco’s modified Eagle’s medium (043-30085; Wako, Osaka, Japan) supplemented with 10% fetal bovine serum. Drp1 KO MEFs^6^ and Mff KO HeLa cells^16^ were generated previously. Mff KO MEFs were generated as described in Supplementary Methods. Lipofectamine LTX (Life Technologies, Carlsbad, CA) and OptiMEM (Gibco) were used for the transfection of poly(I:C)-LMW, poly(I:C)-HMW (#tlrl-picw, #tlrl-pic; InvivoGen, San Diego, CA), and poly(dI:dC) (P4929; Sigma-Aldrich). Lipofectamine LTX with PLUS or Lipofectamine 2000 (Life Technologies) was used for the transfection of plasmid DNA. An empty vector was used to maintain equal amounts of DNA among wells (100 ng in Fig. 1m, 1.25 μg in Fig. 3). Lipofectamine RNAiMAX (Life Technologies) was used for RNAi and small interfering RNA (siRNA) targeting human Mff (siGENOME siRNA SMARTpool, M-018261-01-0005), human MAVS (siGENOME siRNA SMARTpool, M-024237-02-0005), and a non-targeting control siRNA #2 (D-001210-02-05) were purchased from Dharmacon (Lafayette, CO). Other siRNAs are shown in Supplementary Methods. Cells were transfected with 20 nM siRNA and the medium was replaced after 4 h. At 72 h post-transfection, the cells were stimulated or analyzed. Knockdown efficiency was determined by immunoblot analysis.

### Plasmids

Hemagglutinin (HA)-tagged human MAVS pcDNA3.1 was reported previously^26^. FLAG-tagged mouse Mff (WT), Mff (ΔC20), and Mff (ΔN100) pcDNA3.1 were reported previously^13^. Full-length Mff used in this study corresponds to isoform 4 (NM_001310699.1 in NCBI), Mff-209 (ENSMUST00000160972.7 in Ensemble genome), and splice isoform 8 of Mff^13^. pSu9(1-69)-RFP (mitoRFP) was reported previously^55^.

### RNA virus infection

SeV Cantell strain was purchased from ATCC (VR-907; Manassas, VA)^56^. MEFs were infected with SeV [4 HA units/mL] for 10 and 24 h, and the cell-free supernatants were analyzed by ELISA to measure the amount of IFN-β.

### Construction of plasmids and Mff KO MEFs stably expressing Mff constructs

FLAG-tagged Mff constructs^13^ were subcloned into the *Bam*HI and *Not*I sites of the pMXs-IP vector^57^. For the phosphorylation-mimic or phosphorylation-negative mutant of Mff, S146 (TCC) of Mff was changed to D (GAC) or A (GCC), respectively. We also constructed S146E (TCC to GAG) of Mff mutant for another phosphorylation-mimic mutant. The primers are listed in Supplementary Table 1. Mutagenesis was carried out using a PrimeSTAR Mutagenesis Basal Kit (Takara Bio, Inc., Shiga, Japan) and confirmed by sequencing. Mff KO MEFs were transfected with empty or each expression pMXs-IP vector (1.25 μg) in a 6-well format by Lipofectamine 2000, the medium was replaced at 4 h, and the cells were further cultured for 24–48 h. All stable cell lines were established by subsequent selection in the presence of puromycin (2 μg/mL) for approximately 10 days and non-transfected parental cells were confirmed to be dead.

### Chemicals and antibodies

AMPK activator AICAR (2 mM, ab120358) and AMPK inhibitor compound C (20 μM, ab120843) were purchased from Abcam. Respiratory inhibitors rotenone (0.5 μM) or antimycin A (1 μM) and ATP synthase inhibitor oligomycin (1 μM) were purchased from Sigma-Aldrich. MitoTracker Red CMXRos (100 nM, M7512) and MitoTracker Deep Red FM (100 nM, M22426) were purchased from Molecular Probes. Final concentration of dimethyl sulfoxide was set to 0.5% during the treatment with compound C, rotenone, antimycin A, or oligomycin. The primary and secondary antibodies used for immunoblotting or immunostaining are listed in Supplementary Table 2. Additional information of materials and antibodies are shown in Supplementary Methods.

### Quantitative real-time reverse transcriptase-PCR (RT-PCR)

Total RNA was isolated from cells using Sepasol RNA I Super G (Nacalai Tesque, Kyoto, Japan), and cDNA synthesis was performed using a QuantiTect Reverse Transcription Kit (Qiagen, Hilden, Germany). cDNAs were quantified with gene-specific primer pairs (Supplementary Table 1) using TB Green Premix Ex Taq II (Tli RNaseH Plus; Takara) and quantitative real-time RT-PCR was conducted using the ABI7500 system or StepOnePlus (Applied Biosystems, Foster City, CA). Gene expression values were normalized to glyceraldehyde-3-phosphate dehydrogenase (*Gapdh*) gene expression using the delta-delta Ct method. For relative gene expression (fold), the control sample in the left-most column of each bar graph was standardized at a value of 1.

### Enzyme-linked immunosorbent assay

MEFs were transfected with poly(I:C)-LMW (1 μg/mL), poly(I:C)-HMW (1 μg/mL), or poly(dI:dC) (1 μg/mL), or stimulated with LPS (100 ng/mL) or bovine serum albumin (BSA)-conjugated palmitate (800 μM) for 8 h. The culture supernatants were collected and centrifuged to remove nonadherent cells. The concentrations of mouse IFN-β (#42400-1; PBL Assay Science, Piscataway, NJ, or MIFNB0; R&D Systems, Minneapolis, MN) and mouse IL-6 (M6000B; R&D Systems) were determined by ELISA according to the manufacturers’ instructions. The supernatants of SeV-infected MEFs were also examined.

### Immunoblotting

The cells were collected at each indicated time point following stimulation. The cells were washed with ice-cold phosphate-buffered saline (PBS) twice, and lysed on ice in lysis buffer (20 mM Tris-HCl pH 7.6, 150 mM NaCl, 2 mM EDTA, 0.5% NP-40) with a protease inhibitor cocktail tablet and a phosphatase inhibitor cocktail tablet (Roche Molecular Biochemicals, Mannheim, Germany). The lysates were sonicated and clarified by centrifugation at 16,500 × *g* at 4 °C for 10 min. Equal amount protein samples were added to 2× Laemmli sample buffer (#161-0737; Bio-Rad, Hercules, CA) with 2-mercaptoethanol (2-ME) or 4× sample buffer (0.25 M Tris-HCl pH 6.8, 4% glycerol, 8% SDS, 0.008% bromophenol blue) with 20% 2-ME and boiled at 98 °C for 5 min. The samples were separated by SDS-PAGE and transferred to a polyvinylidene difluoride membrane (GE Healthcare or Immobilon-P from Millipore, Burlington, MA). Primary and secondary antibodies were diluted in Can Get Signal Solution 1 and 2 (Toyobo Co., Ltd., Osaka, Japan) or TBST containing 5% BSA or 5% non-fat dry milk as shown in Supplementary Table 2. Detection was carried out with ECL (RPN2106, GE Healthcare) or Immobilon Western Chemiluminescent HRP Substrate (Millipore) using LAS4000 mini (GE Healthcare) or VersaDoc 5000 (Bio-Rad). Images were analyzed using ImageJ (National Institutes of Health, Bethesda, MD) or ImageQuant (GE Healthcare). Image brightness and contrast were adjusted by ImageQuant or Photoshop CC (Adobe).

### Immunostaining and confocal microscopic analysis

The cells were seeded into four-well chamber slides or on coverslips. To visualize mitochondrial network, cells were transiently expressed with mitoRFP (250 ng) or stained with MitoTracker Red CMXRos or MitoTracker Deep Red for 15–30 min at 37 °C under conditions of 5% CO_2_. The cells were fixed in 4% paraformaldehyde for 15 min at room temperature and permeabilized with 0.1% Triton X-100 for 5 min at room temperature. After blocking with PBS containing 5% non-fat dry milk for 1 h, the cells were stained with primary antibodies for 1 h at room temperature or overnight at 4 °C. After washing with PBS, the cells were incubated with Alexa Fluor-labeled secondary antibodies (1:500) for 1 h at room temperature. Primary and Alexa Fluor-labeled secondary antibodies were diluted in PBS containing 5% non-fat dry milk or Can Get Signal immunostain (Toyobo) as listed in Supplementary Table 2. After incubation with DAPI for 5 min or Hoechst 33342 for 10 min, the cells were washed with PBS and mounted with Fluoromount (Diagnostic BioSystems, Inc., Pleasanton, CA) or SlowFade Gold (Invitrogen). Images (1024 × 1024 pixels) were acquired using a LSM700 (Zeiss, Oberkochen, Germany) using ×63/1.4 oil-immersion lens. For Fig. 7c, images were acquired with ×20 objective. The image data were analyzed using ZEN 2010 (Zeiss). *Z*-stacked images (*z* = 0.25–0.37 μm, 5–9 images) were projected in Figs. 2a–c, 3e, f, 4d, 5f, and 7a and Supplementary Figs. 1c–d, f, 3, and 4h.

For live-cell imaging, the cells were seeded in a collagen type I-coated 35-mm glass-based dish (4970-011, Iwaki; Asahi Glass Co., Ltd., Tokyo, Japan) and stained for 15 min at 37 °C with MitoTracker Red CMXRos and Hoechst 33342 as needed under conditions of 5% CO_2_. After washing with culture medium three times, the cells were rested for 30 min in culture medium containing 50 mM HEPES under 37 °C and then transfected with poly(I:C)-LMW as needed. Live-cell images (1024 × 1024 pixels) were acquired using a LSM700 using ×63/1.4 oil-immersion lens under 37 °C, and the images were processed using ZEN 2010. *Z*-stacked images (*z* = 0.37 μm, 3–4 images) were projected in Fig. 5e and Supplementary Figs. 1e and 4a, g, j.

For quantification of MAVS distribution in fluorescent images, we measured co-localization efficiency between mitochondria and MAVS. The double-staining images (512 × 512 pixels) of mitochondria and MAVS were acquired using a LSM700 and ZEN 2010 program (Zeiss) with a ×63/1.4 oil-immersion lens. The threshold of MAVS signal was set as half of the highest intensity in the image, and threshold of mitochondrial marker was set using Otsu’ auto threshold algorism in Fiji/ImageJ program. Using these double-staining binary images of MAVS and mitochondrial marker, the ratio of MAVS signal overlapped on mitochondria was measured by co-localization application in ZEN 2010. In Fig. 2e, co-localization ratio between MAVS and Mff or Drp1 in WT MEFs was calculated using each of binary threshold image by Otsu’ auto threshold algorism. For analysis of cytoplasmic MAVS, the percentage of cells with cytoplasmic MAVS (MAVS signal without MitoTracker Red) was counted (>800 cells per group).

### Statistics and reproducibility

Data are presented as means ± standard error of the mean of three independent experiments unless otherwise noted. Each *n* value basically shows biological replicates and repeated measurement was considered to be technical replicates. In quantification of fluorescent images, region of interest was counted as *n* value. Images are representative results from basically three independent experiments with similar results. Results were analyzed statistically using a two-tailed unpaired Student’s *t* test or Mann–Whitney’s *U* test (0 h in Fig. 1i, j). A *P* value <0.05 was considered statistically significant. Statistical analysis was carried out using Statcel4 (OMS). No statistical method was used to predetermine sample size. Samples were blindly and randomly divided to experimental groups. The investigators were not blinded to allocation during the experiments and outcome assessment.

### Reporting summary

Further information on research design is available in the Nature Research Reporting Summary linked to this article.

## Supplementary information

Supplementary Information

Reporting Summary

## Data Availability

Data supporting the findings of this study are available in the article and its Supplementary files or from the corresponding author upon reasonable request. Source data are provided with this paper.

## Source data

Source Data
