# Supplementary material for: MAVS is energized by Mff which senses mitochondrial metabolism via AMPK for acute antiviral immunity
Source: Nat Commun. 2020 Nov 11;11:5711. doi: 10.1038/s41467-020-19287-7 (PMC7658986; doi:10.1038/s41467-020-19287-7)
Supplement: Supplementary file 1 — Supplementary Information [file 41467_2020_19287_MOESM1_ESM.pdf]

Supplementary Information

MAVS is energized by Mff which senses mitochondrial metabolism via AMPK for acute antiviral immunity

Yuki Hanada, Naotada Ishihara*, Lixiang Wang, Hidenori Otera, Takaya Ishihara, Takumi Koshiba, Katsuyoshi Mihara, Yoshihiro Ogawa, Masatoshi Nomura

This file contains:
Supplementary Figures 1–4
Supplementary Tables 1 and 2
Supplementary Methods
Supplementary References

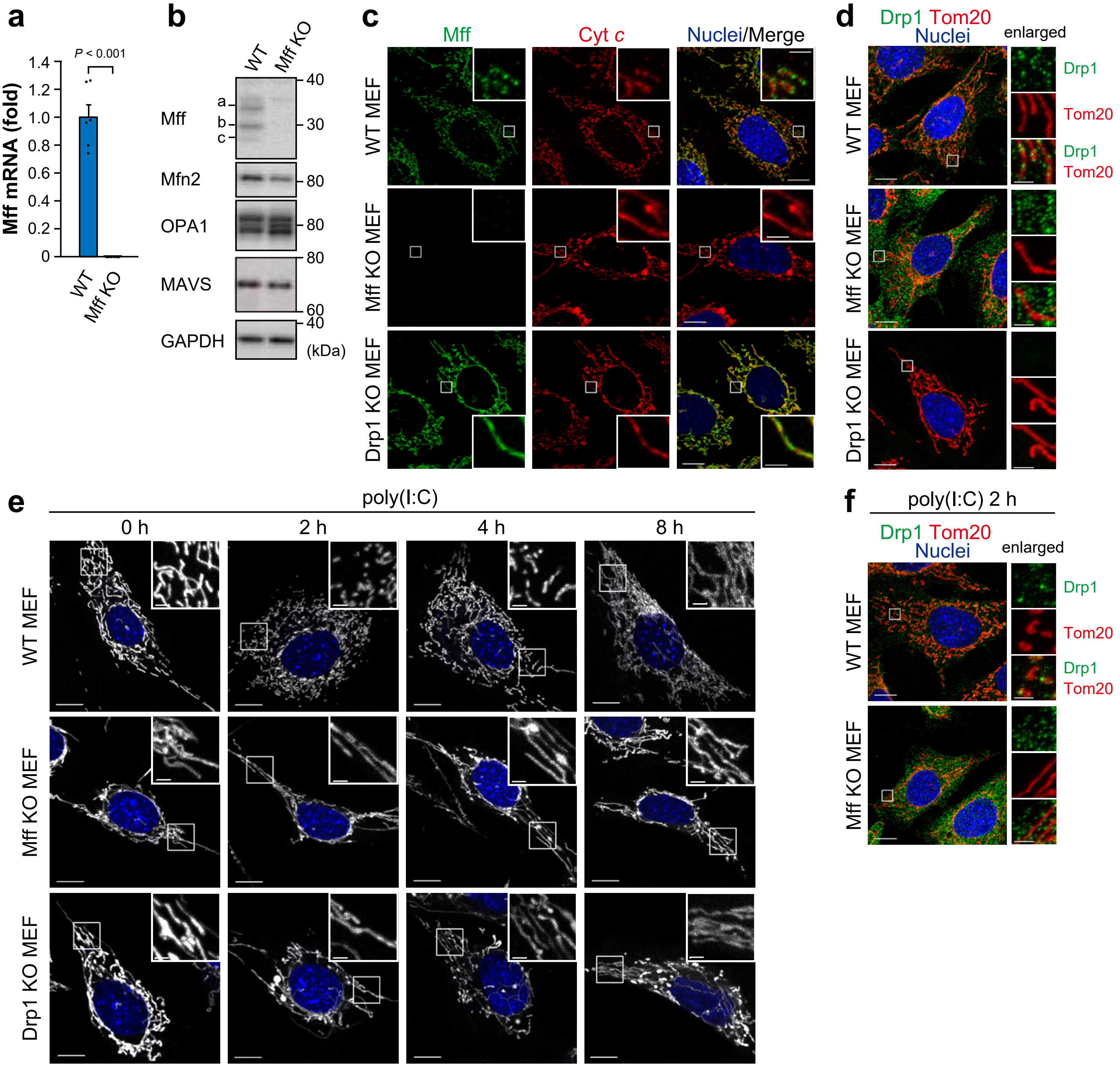

Supplementary Figure 1. Viral RNA induces Mff-dependent transient mitochondrial fission

a, qRT-PCR analysis of Mff mRNA in WT and Mff KO MEFs. Data are means ± SEM of three independent experiments (n=6). $P < 0.001$; two-tailed unpaired *t*-test. **b,** Immunoblot analyses in WT and Mff KO MEFs using antibodies against the indicated mitochondrial dynamics proteins and MAVS. The indicated letters (a, b, c) correspond to the endogenous Mff bands observed in WT MEFs. **c,** Confocal images of Mff expression and mitochondrial morphology in MEFs analyzed by immunofluorescence staining with an anti-Mff antibody (green) and anti-cytochrome *c* (Cyt *c*) antibody (red). Nuclei were stained with Hoechst 33342 (blue). Scale bars: 10 µm (whole) and 2 µm (inset). **d,** Confocal images of Drp1 recruitment to mitochondria. WT, Mff KO, and Drp1 KO MEFs were immunostained with an anti-Tom20 antibody (red) and anti-Drp1 antibody (green). Scale bars: 10 µm (whole) and 2 µm (magnified). Nuclei were stained with Hoechst 33342 (blue). **e,** Representative live-cell images of MitoTracker Red (mitochondria; white) during antiviral response. WT MEFs, Mff KO MEFs, and Drp1 KO MEFs were transfected with poly(I:C)-LMW (1 µg/mL) for the indicated times. A magnified view of the boxed region is shown as an inset. Scale bars: 10 µm (whole) and 2 µm (inset). Nuclei were stained with Hoechst 33342 (blue). **f,** WT and Mff KO MEFs with transfected poly(I:C)-LMW (1 µg/mL) for 2 h were immunostained with an anti-Tom20 antibody (red) and anti-Drp1 antibody (green). Scale bars: 10 µm (whole) and 2 µm (magnified). Nuclei were stained with Hoechst 33342 (blue). Representative images from three independent experiments are shown in **b**–**f**.

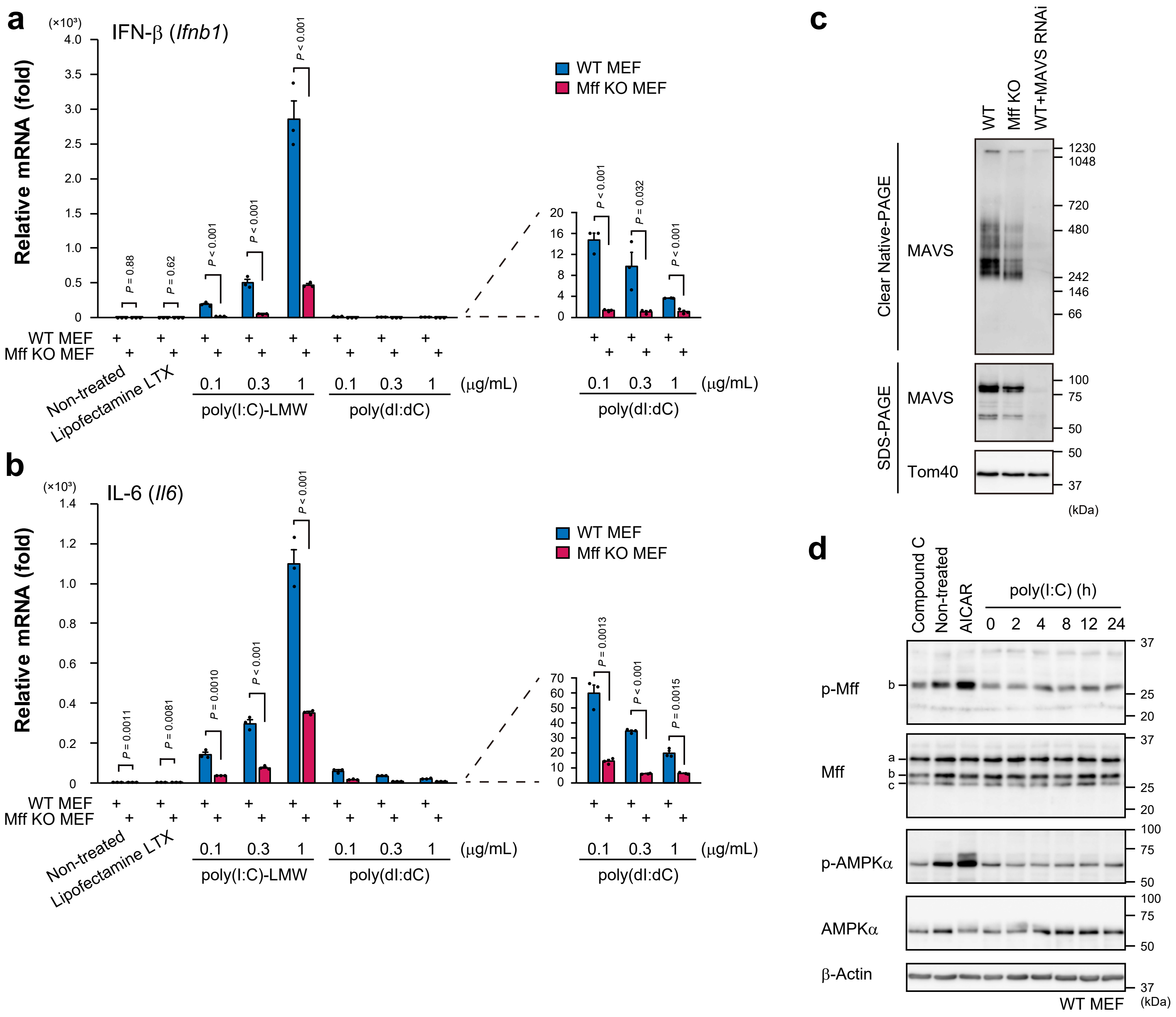

Supplementary Figure 2. MAVS oligomeric status and AMPK-Mff activity in antiviral response
a, b, qRT-PCR analyses of IFN-β (**a**) and IL-6 (**b**) mRNA in WT and Mff KO MEFs transfected with increase amount of poly(I:C)-LMW or poly(dI:dC) (0.1, 0.3, 1 μg/mL) for 8 h. Same amount of Lipofectamine LTX was used for transfection. Data are means ± SEM of one experiment (n=3) and another independent experiment was performed with similar results. *P* values; two-tailed unpaired *t*-test. **c,** Clear-native PAGE (CN-PAGE) of MAVS. Mitochondrial fraction from WT and Mff KO HeLa expressing MAVS, and WT HeLa cells with MAVS siRNA were subjected to CN-PAGE and 10% input was subjected to SDS-PAGE and detected by anti-MAVS or anti-Tom40 antibodies. The experiment was repeated independently two times with similar observation. **d,** Immunoblot analyses of Mff and AMPK phosphorylation in WT MEFs during antiviral response. Cells were transfected with poly(I:C)-LMW (1 μg/mL) for the indicated times. AICAR (2 mM) or compound C (20 μM) was used as an AMPK activator or inhibitor, respectively. The indicated letters (a, b, c) correspond to the endogenous Mff bands observed in WT MEFs. Representative images are shown from three independent experiments in **d**.

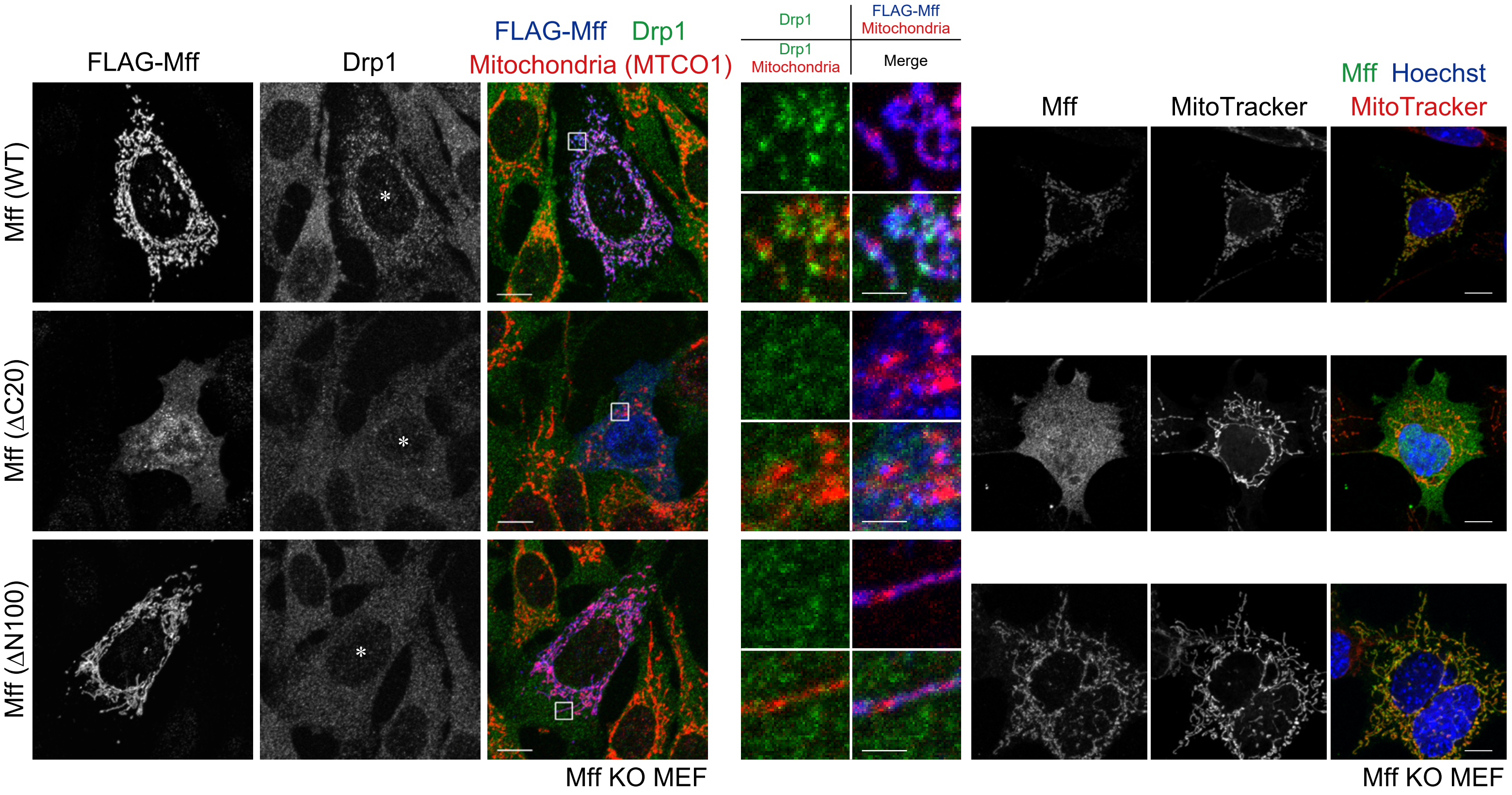

Supplementary Figure 3. Drp1 localization in Mff KO MEFs expressed with truncated Mff constructs
Representative confocal microscopic images of Drp1 localization and mitochondrial morphology. (Left panels) Mff KO MEFs transfected with expression vectors for FLAG-tagged Mff (WT), Mff (ΔC20), or Mff (ΔN100). At 24 h after transfection, the cells were immunostained with an anti-FLAG antibody (rabbit IgG, blue), an anti-Drp1 antibody (mouse IgG1, green) and an anti-MTCO1 antibody (mouse IgG2a, red) as a mitochondrial marker. Asterisks (*) show the expressed cells. A magnified view of the boxed region is shown in the right panel. Scale bars: 10 µm (whole) and 2 µm (magnified). (Right panels) Similar to the left panels, Mff KO MEFs transiently expressing Mff mutants were stained with MitoTracker Deep Red and immunostained with an anti-Mff antibody. Scale bars: 10 µm. Experiments in this figure were repeated at least two times independently with similar results.

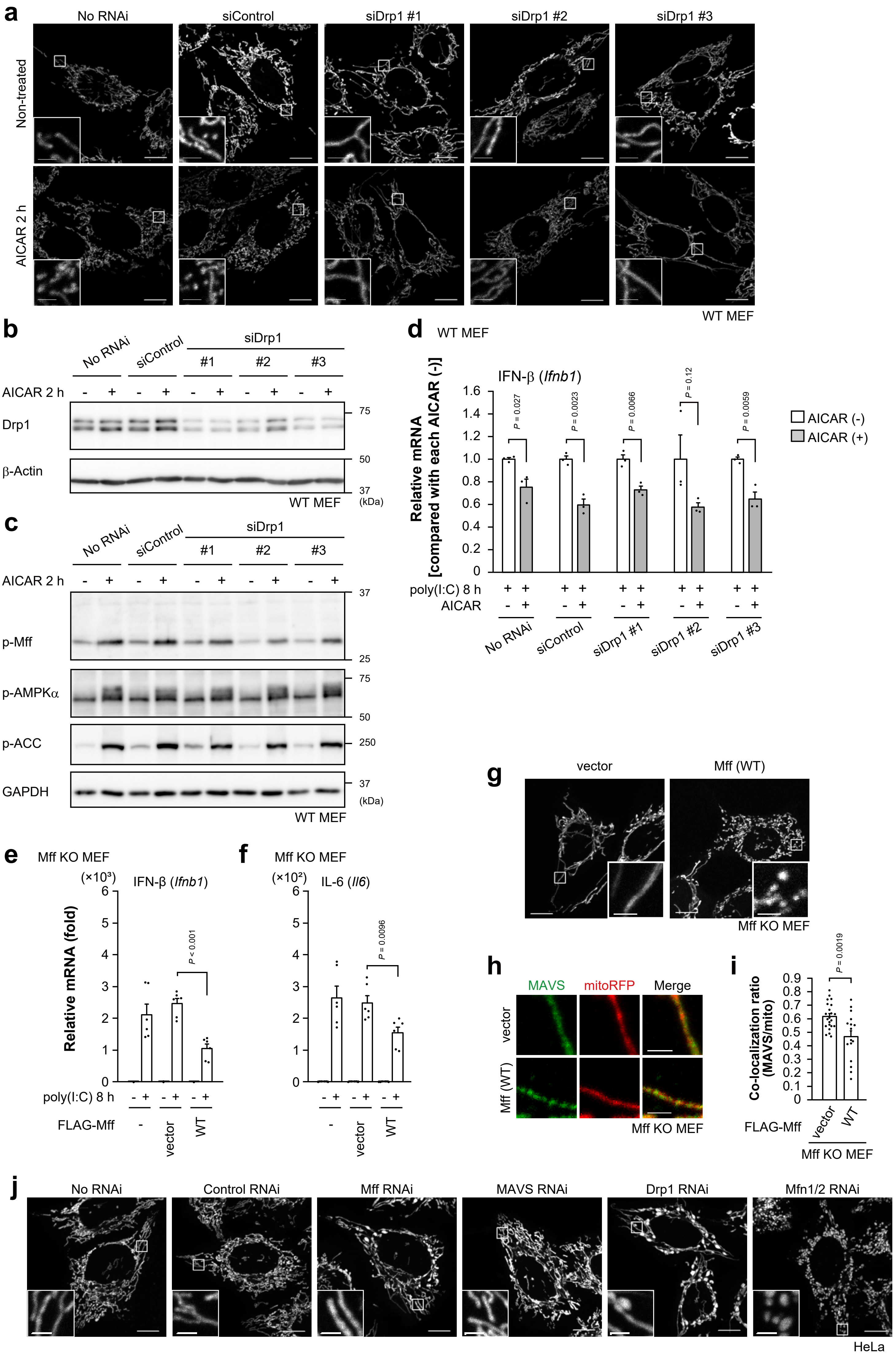

a
No RNAi
siControl
siDrp1 #1
siDrp1 #2
siDrp1 #3
Non-treated
AICAR 2 h
WT MEF
b
No RNAi
siControl
siDrp1
#1
#2
#3
AICAR 2 h
Drp1
β-Actin
75
50
37
(kDa)
WT MEF
c
p-Mff
p-AMPKα
p-ACC
GAPDH
37
25
75
50
250
37
(kDa)
WT MEF
d
WT MEF
IFN-β (Ifnb1)
Relative mRNA [compared with each AICAR (-)]
P = 0.027
P = 0.0023
P = 0.0066
P = 0.12
P = 0.0059
AICAR (-)
AICAR (+)
poly(I:C) 8 h
AICAR
No RNAi
siControl
siDrp1 #1
siDrp1 #2
siDrp1 #3
e
Mff KO MEF
(×10³)
IFN-β (Ifnb1)
Relative mRNA (fold)
P < 0.001
poly(I:C) 8 h
FLAG-Mff
vector
WT
f
Mff KO MEF
(×10²)
IL-6 (Il6)
P = 0.0096
vector
WT
g
vector
Mff (WT)
Mff KO MEF
h
MAVS
mitoRFP
Merge
vector
Mff (WT)
Mff KO MEF
i
Co-localization ratio (MAVS/mito)
P = 0.0019
FLAG-Mff
vector
WT
Mff KO MEF
j
No RNAi
Control RNAi
Mff RNAi
MAVS RNAi
Drp1 RNAi
Mfn1/2 RNAi
HeLa

Supplementary Figure 4. Regulation of the antiviral response by Mff is independent of mitochondrial morphology
a, Representative live-cell images of MitoTracker Red (mitochondria; white) in WT MEFs without RNAi or transfected with control or Drp1 siRNA (#1–3) in the absence or presence of AICAR (2 mM) for 2 h. Scale bars: 10 µm (whole) and 2 µm (inset). Images were from two independent experiments. **b, c,** Immunoblot analyses of cell lysates in (**a**) using antibodies against the indicated proteins. Images were from two independent experiments. **d,** qRT-PCR analysis of IFN-β mRNA in WT MEFs with Drp1 RNAi transfected with poly(I:C)-LMW (1 µg/mL) in the absence (−) or presence (+) of AICAR (2 mM, pre-treated for 2 h) for 8 h. Induction of IFN-β mRNA in AICAR (+) was compared with that in AICAR (−). Data represent means ± SEM of one experiment (n=3) and reproducibility of the results was confirmed by another independent experiment. *P* values; two-tailed unpaired *t*-test. **e, f,** qRT-PCR of IFN-β (**e**) and IL-6 (**f**) mRNA in Mff KO MEFs stably expressing Mff (WT) with or without poly(I:C)-LMW (1 µg/mL) transfection for 8 h. Data represent means ± SEM of three independent experiments (n=6). The values in Mff KO MEFs and Mff KO MEFs stably expressing empty vector are repeatedly presented in Fig. 5c and 5d. *P* values; two-tailed unpaired *t*-test. **g,** Representative live-cell images of MitoTracker Red (mitochondria, white) in Mff KO MEFs stably expressing Mff (WT) or empty vector. Scale bars: 10 µm (whole) and 2 µm (inset). Experiments were repeated three times independently with similar results. **h, i,** Magnified images of MAVS on mitochondria in Mff KO MEFs stably expressing Mff (WT) or empty vector (**h**). The cells were transiently transfected with a mitochondrial marker mitoRFP (red) and immunostained with an anti-MAVS antibody (green). Scale bars: 2 µm. Co-localization ratio between MAVS and mitoRFP in Mff KO MEFs stably expressing Mff (WT) or empty vector (**i**). Data represent means ± SEM of three independent experiments (vector, n=22; WT, n=16). The values in Mff KO MEFs stably expressing empty vector are repeatedly presented in Fig. 5g. *P* value; two-tailed unpaired *t*-test. **j,** Representative live-cell images of MitoTracker Red (mitochondria; white) in HeLa cells without RNAi or transfected with control, Mff, MAVS, Drp1 or Mfn1/2 siRNA and cultured for 72 h. Scale bars: 10 µm (whole) and 2 µm (inset). Experiments were repeated two times with similar results.

Supplementary Table 1. Primers used in this study

For	Name	Forward	Reverse	Product size (bp)
qRT-PCR	Mouse *Ifnb1*	5'-CCCTATGGAGATGACGGAGA-3'	5'-CTGTCTGCTGGTGGAGTTCA-3'	161
qRT-PCR	Mouse *Il6*	5'-CCGGAGAGGAGACTTCACAG-3'	5'-TTCTGCAAGTGCATCATCGT-3'	166
qRT-PCR	Mouse *Ifna4*	5'-TCCATCAGCAGCTCAATGAC-3'	5'-TATGTCCTCACAGCCAGCAG-3'	88
qRT-PCR	Mouse *Mff*	5'-GGAGTTCCAAATGCCAGTGT-3'	5'-TGGTGTTTTCAGTGCCAGAG-3'	144
qRT-PCR	Mouse *Gapdh*	5'-CCATCACTGCCACCCAGAAG-3'	5'-GATGCAGGGATGATGTTC-3'	91
qRT-PCR	Human *IFNB1*	5'-TGCTCTGGCACAACAGGTAG-3'	5'-CAGGAGAGCAATTTGGAGGA-3'	82
qRT-PCR	Human *GAPDH*	5'-CCATCACTGCCACCCAGAAG-3'	5'-AGAGGCAGGGATGATGTTCT-3'	92

For	Name	Sequence	Details
Site direct mutagenesis	mMff S146D-F	5'-AACGATGACATGTATGGCATTTCAAAC-3'	5'-Oligo for mMff S146D
Site direct mutagenesis	mMff S146D-R	5'-ATACATGTCATCGTTTCTGACCAACTG-3'	3'-Oligo for mMff S146D
Site direct mutagenesis	mMff S146E-F	5'-AACGATGAGATGTATGGCATTTCAAAC-3'	5'-Oligo for mMff S146E
Site direct mutagenesis	mMff S146E-R	5'-ATACATCTCATCGTTTCTGACCAACTG-3'	3'-Oligo for mMff S146E
Site direct mutagenesis	mMff S146A-F	5'-AACGATGCCATGTATGGCATTTCAAAC-3'	5'-Oligo for mMff S146A
Site direct mutagenesis	mMff S146A-R	5'-ATACATGGCATCGTTTCTGACCAACTG-3'	3'-Oligo for mMff S146A
Subcloning	N-FLAG-mMff	5'-GCGGATCCATGGACTACAAAGACGATGACG-3'	5'-Oligo for N-FLAG-mMff with BamH I , ATG and FLAG-tag
Subcloning	mMff	5'-CTGCGGCCGCCTAGCGTCGAAAC-3'	3'-Oligo for mMff with Not I
Subcloning	mMffΔC	5'-CGGCGGCCGCCTACATTTCTC-3'	3'-Oligo for mMffΔC with stop codon and Not I

Supplementary Table 2. Antibodies used in this study

Antibodies	Dilution for immunoblotting	Source	Identifier
Rabbit polyclonal anti-human MAVS	1:1000	Cell Signaling Technology	#3993
Rabbit polyclonal anti-rodent specific MAVS	1:1000 in CanGet signal 1	Cell Signaling Technology	#4983
Rabbit monoclonal anti-phospho-IRF3 Ser396 (4D4G)	1:1000 in CanGet signal 1	Cell Signaling Technology	#4947
Rabbit monoclonal anti-IRF3 (D83B9)	1:5000 in CanGet signal 1	Cell Signaling Technology	#4302
Rabbit monoclonal anti-phospho-TBK1 Ser172 (D52C2)	1:2000 in CanGet signal 1	Cell Signaling Technology	#5483
Rabbit polyclonal anti-TBK1	1:5000 in CanGet signal 1	Cell Signaling Technology	#3013
Rabbit monoclonal anti-phospho-NFκB p65 Ser536 (93H1)	1:1000 in 5%BSA/TBST	Cell Signaling Technology	#3033
Rabbit polyclonal anti-phospho-Mff Ser146	1:1000 in 5%BSA/TBST	Cell Signaling Technology	#49281
Rabbit polyclonal anti-Mff	1:1000	Proteintech	17090-1-AP
Rabbit polyclonal anti-phospho-Drp1 Ser616	1:1000 in CanGet signal 1	Cell Signaling Technology	#3455
Mouse monoclonal anti-Drp1 (clone 8)	1:1000	BD Biosciences	611113
Rabbit polyclonal anti-Mfn1 (H-65)	1:1000 in CanGet signal 1	Santa Cruz	sc-50330
Mouse monoclonal anti-Mfn2 (M03, clone 4H8)	1:1000 in CanGet signal 1	Abnova	H00009927-M03
Mouse monoclonal anti-OPA1 (clone 18)	1:1000	BD Biosciences	612606
Rabbit monoclonal anti-phospho-AMPKα Thr172 (40H9)	1:1000 in 5%BSA/TBST	Cell Signaling Technology	#2535
Rabbit polyclonal anti-AMPKα	1:1000	Cell Signaling Technology	#2532
Rabbit polyclonal anti-phospho-ACC Ser79	1:1000 in 5%BSA/TBST	Cell Signaling Technology	#3661
Mouse monoclonal anti-β-actin (AC-74)	1:10000	Sigma-Aldrich	A2228
Rabbit monoclonal anti-GAPDH (14C10)	1:1000	Cell Signaling Technology	#2118
Peroxidase-conjugated monoclonal anti-GAPDH	1:5000	Wako	015-25473
Rabbit polyclonal anti-VDAC1/Porin	1:5000	Abcam	ab15895
Rabbit polyclonal anti-Tom40	1:1000	Proteintech	18409-1-AP
Mouse monoclonal anti-FLAG M2	1:1000	Sigma-Aldrich	F3165
Mouse monoclonal anti-FLAG M2	1:1000	Sigma-Aldrich	F1804
Anti-mouse IgG, HRP-linked antibody	1:5000	Cell Signaling Technology	#7076
Anti-rabbit IgG, HRP-linked antibody	1:5000	Cell Signaling Technology	#7074
Anti-mouse IgG, HRP-linked antibody	1:2000-1:10000	Invitrogen	G21040
Anti-rabbit IgG, HRP-linked antibody	1:2000	Invitrogen	G21234

Antibodies	Dilution for immunostaining	Source	Identifier
Rabbit polyclonal anti-rodent specific MAVS	1:200 in CanGet signal A	Cell Signaling Technology	#4983
Mouse monoclonal anti-MAVS (C-1)	1:200	Santa Cruz	sc-365333
Rabbit polyclonal anti-Mff	1:200	Proteintech	17090-1-AP
Mouse monoclonal anti-Drp1 (clone 8)	1:500	BD Biosciences	611113
Mouse monoclonal anti-Cyt *c* (6H2.B4)	1:500	BD Biosciences	556432
Rabbit polyclonal anti-Tom20 (FL-145)	1:500	Santa Cruz	sc-11415
Mouse monoclonal anti-MTCO1 (1D6E1A8)	1:50	BioSciences (Abcam)	ab14705
Mouse monoclonal anti-FLAG M2	1:200 in CanGet signal A	Sigma-Aldrich	F3165
Mouse monoclonal anti-FLAG M2	1:200	Sigma-Aldrich	F1804
Rabbit polyclonal anti-FLAG	1:500	Sigma-Aldrich	F7425
Alexa Fluor 488 goat anti-rabbit IgG	1:500	Invitrogen	A11034
Alexa Fluor 568 goat anti-rabbit IgG	1:500	Invitrogen	A11011
Alexa Fluor 488 goat anti-mouse IgG	1:500	Invitrogen	A11001
Alexa Fluor 568 goat anti-mouse IgG	1:500	Invitrogen	A11004
Alexa Fluor 647 goat anti-mouse IgG1	1:500	Invitrogen	A21240
Alexa Fluor 647 goat anti-mouse IgG2a	1:500	Invitrogen	A21241

Antibodies	Dilution for ELISA	Source	Identifier
IFN-β	manufacturer's protocol	PBL Assay Science	42400-1
IFN-β	manufacturer's protocol	R&D Systems	MIFNB0
IL-6	manufacturer's protocol	R&D Systems	M6000B

Supplementary Methods

Generation of Mff KO MEFs

Mff KO MEFs were generated as reported previously[1] with a slight modification. Briefly, from Mff lox/lox mice with exons 3 and 4 flanked by loxP (Kato H and Masuda K *et al*., in preparation), MEFs were prepared from E12.5 embryos. Adeno-Cre recombinase or empty vector was expressed in the Mff lox/lox MEFs, and a neomycin-resistant gene was used to select Mff KO MEFs or Mff lox/lox MEFs (WT MEFs).

Clear-native PAGE

Clear-native PAGE (CN-PAGE) was performed as described previously[2,3]. WT and Mff KO HeLa cells transiently expressed HA-tagged MAVS, and WT HeLa cells transfected with MAVS siRNA for 72 h were suspended in homogenization buffer [Hb: 10 mM HEPES-KOH buffer (pH 7.4) containing 0.22 M mannitol, 0.07 M sucrose and protease inhibitor cocktail (Roche)] and mitochondrial fractions were prepared as described previously[3] with 10 strokes and without adding DTT. Equal amount of fresh mitochondrial fractions (6 μg protein in 40 μL) were solubilized with 20 μL of solubilizing buffer [Hb containing 0.75% *n*-Dodecyl-β-D-maltoside (DDM) and 50 mM NaCl] for 1 h on ice. The lysates were clarified by centrifugation at 16,500 × g at 4°C for 30 min. The supernatants were mixed with 10× sample buffer (50% glycerol and 0.1% Ponceau S) and subjected to CN-PAGE using NativePAGE 4-16% Bis-Tris Protein Gels (BN1002BOX; Thermo) followed by immunoblotting with anti-MAVS antibody. Before adding the sample buffer, 10% input of the supernatant were subjected to SDS-PAGE and detected by anti-MAVS or anti-Tom40 antibodies. NativeMark Unstained Protein Standard (LC0725; Thermo) was used for CN-PAGE.

Sequences of RNAi oligonucleotides

Three siRNAs targeting mouse Drp1 were purchased from Nippon gene (Tokyo, Japan) and the target sequences were as follows: 5′-ACCGCAAAGUACAUUGAAA-3′ (#1), 5'-GGGCAGCUGUAUAAGUCAU-3' (#2), and 5'-GCGCAGAACUCUAGCUGUA-3' (#3). The target sequences of others were reported previously as follows: human Drp1[4], 5′-ACUAUUGAAGGAACUGCAAAAUAUA-3′ (siRNA from Bonac); human Mfn1[5], 5′-AUUCCUGUAAUCUUGCAUGAAAUCC-3′ (stealth RNA from Invitrogen); human Mfn2[5], 5′-AAUCCCAGAGGGCAGAACUUUGUCC-3′ (stealth RNA from Invitrogen).

Additional information of material and antibodies

The following materials were used: LPS from *Escherichia coli* O111:B4 (referred to as LPS-B4; Sigma-Aldrich) or *Salmonella enterica* serotype Minnesota Re 595 (referred to as LPS-595; Sigma-Aldrich) (100 ng/mL), bovine serum albumin (BSA)-conjugated palmitate (800 μM; Sigma-Aldrich)[6],

Hoechst 33342 (1 μg/mL, H342; Dojindo Laboratories, Kumamoto, Japan), and DAPI (1 μg/mL, D523; Dojindo). In this study, anti-MAVS antibodies were used as follows: #3993 (CST) in Figs. 1l, 1m, Supplementary Fig. 2c; #4983 (CST) in Figs. 1g, 2b, 2c, 4a, 7b, Supplementary Fig. 1a; C-1 (Santa Cruz) in Figs. 2a, 3e, 3f, 4d, 5f, 7a, Supplementary Fig. 4h.

Supplementary References

1. Ishihara, N., Nomura, M., Jofuku, A., Kato, H., Suzuki, S. O., Masuda, K., Otera, H., Nakanishi, Y., Nonaka, I., Goto, Y., Taguchi, N., Morinaga, H., Maeda, M., Takayanagi, R., Yokota, S., & Mihara, K. Mitochondrial fission factor Drp1 is essential for embryonic development and synapse formation in mice. *Nat. Cell Biol.* **11**, 958–966 (2009).
2. Ban, T., Ishihara, T., Kohno, H., Saita, S., Ichimura, A., Maenaka, K., Oka, T., Mihara, K., & Ishihara, N. Molecular basis of selective mitochondrial fusion by heterotypic action between OPA1 and cardiolipin. *Nat. Cell Biol.* **19**, 856–863 (2017).
3. Ishihara, N., Maeda, M., Ban, T. & Mihara, K. Cell-free mitochondrial fusion assay detected by specific protease reaction revealed Ca^{2+} as regulator of mitofusin-dependent mitochondrial fusion. *J. Biochem.* **162**, 287–294 (2017).
4. Taguchi, N., Ishihara, N., Jofuku, A., Oka, T. & Mihara, K. Mitotic phosphorylation of dynamin-related GTPase Drp1 participates in mitochondrial fission. *J. Biol. Chem.* **282**, 11521–11529 (2007).
5. Ban-Ishihara, R., Ishihara, T., Sasaki, N., Mihara, K. & Ishihara, N. Dynamics of nucleoid structure regulated by mitochondrial fission contributes to cristae reformation and release of cytochrome *c*. *Proc. Natl. Acad. Sci. U. S. A.* **110**, 11863–11868 (2013).
6. Das, S. K., Chu, W. S., Mondal, A. K., Sharma, N. K., Kern, P. A., Rasouli, N., & Elbein, S. C. Effect of pioglitazone treatment on endoplasmic reticulum stress response in human adipose and in palmitate-induced stress in human liver and adipose cell lines. *Am. J. Physiol. Endocrinol. Metab.* **295**, E393–E400 (2008).
