# Supplementary material for: MAVS is energized by Mff which senses mitochondrial metabolism via AMPK for acute antiviral immunity
Source: Nat Commun. 2020 Nov 11;11:5711. doi: 10.1038/s41467-020-19287-7 (PMC7658986; doi:10.1038/s41467-020-19287-7)
Supplement: Supplementary file 2 — Reporting Summary [file 41467_2020_19287_MOESM2_ESM.pdf]

nature research

Corresponding author(s): Naotada Ishihara

Last updated by author(s): Sep 24, 2020

Reporting Summary

Nature Research wishes to improve the reproducibility of the work that we publish. This form provides structure for consistency and transparency in reporting. For further information on Nature Research policies, see Authors & Referees and the Editorial Policy Checklist.

Statistics

For all statistical analyses, confirm that the following items are present in the figure legend, table legend, main text, or Methods section.

n/a	Confirmed	
☐	☒	The exact sample size (*n*) for each experimental group/condition, given as a discrete number and unit of measurement
☐	☒	A statement on whether measurements were taken from distinct samples or whether the same sample was measured repeatedly
☐	☒	The statistical test(s) used AND whether they are one- or two-sided<br>*Only common tests should be described solely by name; describe more complex techniques in the Methods section.*
☒	☐	A description of all covariates tested
☒	☐	A description of any assumptions or corrections, such as tests of normality and adjustment for multiple comparisons
☐	☒	A full description of the statistical parameters including central tendency (e.g. means) or other basic estimates (e.g. regression coefficient) AND variation (e.g. standard deviation) or associated estimates of uncertainty (e.g. confidence intervals)
☐	☒	For null hypothesis testing, the test statistic (e.g. *F*, *t*, *r*) with confidence intervals, effect sizes, degrees of freedom and *P* value noted<br>*Give P values as exact values whenever suitable.*
☒	☐	For Bayesian analysis, information on the choice of priors and Markov chain Monte Carlo settings
☒	☐	For hierarchical and complex designs, identification of the appropriate level for tests and full reporting of outcomes
☒	☐	Estimates of effect sizes (e.g. Cohen's *d*, Pearson's *r*), indicating how they were calculated

Our web collection on statistics for biologists contains articles on many of the points above.

Software and code

Policy information about availability of computer code

Data collection: Fluorescent images were collected using ZEN 2010 (Zeiss). Immunoblotting images were collected using ImageQuant TL v8.1.0.0 (GE Healthcare).

Data analysis: Fluorescent images were analyzed by ZEN 2010 (Zeiss). Auto threshold of fluorescent images was performed by Fiji (ImageJ 1.52p, Java 1.8.0_172, 64 bit). Immunoblotting images were analyzed by ImageQuant TL v8.1.0.0 (GE Healthcare) or Photoshop CC (20.0.5, Adobe), and quantified by ImageJ (1.49v, Java 1.6.0_24, 64 bit). Statistical analyses were performed with Statcel4 (OMS).

For manuscripts utilizing custom algorithms or software that are central to the research but not yet described in published literature, software must be made available to editors/reviewers. We strongly encourage code deposition in a community repository (e.g. GitHub). See the Nature Research guidelines for submitting code & software for further information.

Data

Policy information about availability of data

All manuscripts must include a data availability statement. This statement should provide the following information, where applicable:

- Accession codes, unique identifiers, or web links for publicly available datasets
- A list of figures that have associated raw data
- A description of any restrictions on data availability

Data supporting the findings of this study are available as a Source Data file provided with this paper. Uncropped immunoblotting images associated with Figures and Supplementary Figures are shown in a Source Data file. Additional data during this study are available from the corresponding author upon reasonable request.

Field-specific reporting

Please select the one below that is the best fit for your research. If you are not sure, read the appropriate sections before making your selection.

☒ Life sciences ☐ Behavioural & social sciences ☐ Ecological, evolutionary & environmental sciences

For a reference copy of the document with all sections, see nature.com/documents/nr-reporting-summary-flat.pdf

Life sciences study design

All studies must disclose on these points even when the disclosure is negative.

Sample size	No statistical method was used to predetermine sample size. The sample size was selected based on commonly adopted standards in the field. More than 3 for qRT-PCR. More than 3 for microscopic images.
Data exclusions	No data were excluded from the analysis.
Replication	Experiments reported in this study were basically independently performed two or three times in the same method, and the reproducibility was confirmed. Figs 1h, 1n, 7a, Supplementary Figs 2a-c, and 4a-c were performed more than twice. All attempts at replication were successful.
Randomization	Samples were randomly divided to experimental groups.
Blinding	Samples were blindly and randomly divided to experimental groups.

Reporting for specific materials, systems and methods

We require information from authors about some types of materials, experimental systems and methods used in many studies. Here, indicate whether each material, system or method listed is relevant to your study. If you are not sure if a list item applies to your research, read the appropriate section before selecting a response.

Materials & experimental systems

n/a	Involved in the study	
☐	☒	Antibodies
☐	☒	Eukaryotic cell lines
☒	☐	Palaeontology
☒	☐	Animals and other organisms
☒	☐	Human research participants
☒	☐	Clinical data

Methods

n/a	Involved in the study	
☒	☐	ChIP-seq
☒	☐	Flow cytometry
☒	☐	MRI-based neuroimaging

Antibodies

Antibodies used

All antibodies for immunoblotting and immunostaining are listed in Supplementary Table 2. Antibodies for ELISA are listed in Supplementary Table 2.

Validation

Primary antibodies used in our study were commercially available unless otherwise noted and have been characterized and validated by manufactures as shown below.

Antibodies from Cell Signaling Technology:
MAVS (#3993); Application: WB, IF; Species: human. According to the manufacturer's website, this antibody detects endogenous levels of total human MAVS protein detected at 52 and 75 kDa.
MAVS (#4983); Application: WB, IP, IF; Species: mouse, rat. According to the manufacturer's website, this antibody detects endogenous levels of total MAVS protein (rodent specific).
p-IRF3 (#4947); Application: WB; Species: human, mouse.
IRF3 (#4302); Application: WB, IP; Species: human, mouse, rat, monkey.
p-TBK1 (#5483); Application: WB, IP, IF, Flow Cyt; Species: human, mouse.
TBK1 (#3013); Application: WB, IP; Species: human, mouse, rat, monkey.
p-NFκB p65 (#3033); Application: WB, IP, IF, Flow Cyt; Species: human, mouse, rat, hamster, monkey, pig.
p-Drp1 S616 (#3455); Application: WB, IP, IF, Flow Cyt; Species: human (predicted to react based on 100% sequence homology: mouse, rat, monkey).
p-AMPKα (#2535); Application: WB, IP, IHC; Species: human, mouse, rat, hamster, monkey, D. melanogaster, S. cerevisiae.
AMPKα (#2532); Application: WB, IP; Species: human, mouse, rat, hamster, monkey.
p-ACC (#3661); Application: WB, IP, IHC; Species: human, mouse, rat, monkey.
GAPDH (#2118); Application: WB, IHC, IF, Flow Cyt; Species: human, mouse, rat, monkey, bovine, pig.
p-Mff S146 (#49281); Application: WB; Species: human, mouse, rat. According to the manufacturer's website, this antibody

recognizes endogenous levels of Mff protein only when phosphorylated at Ser146. This antibody has been shown to react with phosphorylated Ser146 of human Mff isoforms 2, 4, and 5 but not phosphorylated Ser172 of human Mff isoform 1 in a published paper cited below. This antibody cross-reacts with a 140 kDa protein of unknown identity. In our experiments, additional multiple bands in WB were observed in MEFs and HeLa cells. We confirmed which band is p-Mff Ser146 protein in WB using AICAR, Compound C, Mff RNAi, or Mff KO cells. This product has been discontinued.
Toyama EQ, Herzig S, Courchet J, et al. Metabolism. AMP-activated protein kinase mediates mitochondrial fission in response to energy stress. Science 351:275-281 (2016).

Antibodies from Proteintech:
Mff (17090-1-AP); Application: WB, IP, IHC, IF, ELISA; Species: human, mouse, rat. According to the manufacturer's website, this antibody recognizes the endogenous Mff protein around 26-29 kDa and 35-38 kDa in WB. In our experiments, additional multiple bands in WB were observed in MEFs and HeLa cells. We confirmed which band is Mff protein in WB and specificity of fluorescent signal in IF using Mff RNAi or Mff KO cells.
Tom40 (18409-1-AP); Application: WB, IP, IHC, IF, FC, ELISA; Species: human, mouse, rat.

Antibodies from BD Biosciences:
Drp1 (611113); Application: WB, IF; Species: human, mouse, rat, dog.
OPA1 (612606); Application: WB, IF; Species: human, mouse, rat, dog, chicken.
Cyt c (556432); Application: IP, Bioimaging, (WB: not recommended); Species: human, mouse, rat.

Antibodies from Santa Cruz Biotechnology:
Mfn1 (sc-50330); Application: WB, IP, IHC, IF, ELISA; Species: human, mouse, rat. This product has been discontinued.
Tom20 (sc-11415); Application: WB, IP, IHC, IF, ELISA; Species: human, mouse, rat. This product has been discontinued.
MAVS (sc-365333); Application: WB, IP, IF, ELISA; Species: mouse. Validation with IF in a published paper cited below.
Hwang MS, Boulanger J, Howe JD, et al. MAVS polymers smaller than 80 nm induce mitochondrial membrane remodeling and interferon signaling. FEBS J. 286: 1543-1560 (2019).

Antibodies from Sigma-Aldrich:
FLAG M2 (F3165); Application: WB, IP, IF etc.
FLAG M2 (F1804); Application: WB, IP, IF etc.
FLAG (F7425); Application: WB, IP, IF.
β-actin (A2228); Application: WB, IHC, IF; Species: human, mouse, rat etc.

Others:
Mfn2 (H00009927-M03, Abnova); Application: WB, IHC, ELISA; Species: human, rat. In addition, we detected Mfn2 protein in MEFs in WB using Can Get Signal (Toyobo), and confirmed the specificity by Mfn2-RNAi.
HRP-conjugated GAPDH (015-25473, Wako); Application: WB; Species: human, mouse, donkey.
VDAC1/Porin (ab15895, Abcam); Application: WB, ICC, IF; Species: human, mouse, rat, dog, zebrafish, chinese hamster.
MTCO1 (ab14705, Abcam); Application: WB, IHC-P, Flow Cyt; Species: human, mouse, rat, cow, Caenorhabditis elegans, zebrafish, rhesus monkey. IF in a published paper cited below.
Banati RB, Middleton RJ, Chan R, et al. Positron emission tomography and functional characterization of a complete PBR/TSPO knockout. Nat Commun. 5:5452 (2014).

Antibodies used in ELISA assays (validated by manufacturers):
IFN-β (plate coated with antibody, part# SMP028; antibody, part# SMP211-1 in 42400-1 from PBL Assay Science)
This assay recognizes mouse IFN-β and no cross-reactivity was detected against the factors listed below by the manufacturer.
human IFN-α, IFN-γ, IFN-κ, IFN-β; rat IFN-α, IFN-β, IFN-γ; mouse IFN-α, IFN-γ; feline IFN-α; pig IFN-α

IFN-β (monoclonal antibody specific for mouse IFN-β, part# 898523 in MIFNB0 from R&D Systems)
IFN-β (monoclonal antibody specific for mouse IFN-β conjugated to HRP, part# 898524 in MIFNB0 from R&D Systems)
This assay recognizes natural and recombinant mouse IFN-β and no significant cross-reactivity or interference was observed against the factors listed below by the manufacturer.
Recombinant mouse: IFN-α1, IFN-α/β R2, IFN-κ, IFN-γ, IL-6, IL-28A, Limitin
Recombinant human: IFN-β1
Recombinant mouse IFN-α/β R1 does not cross-react in this assay but does interfere at concentrations > 5 ng/mL in the assay by the manufacturer.

IL-6 (monoclonal antibody specific for mouse IL-6, part# 892369 in M6000B from R&D Systems)
IL-6 (polyclonal antibody against mouse IL-6 conjugated to HRP, part# 892665 in M6000B from R&D Systems)
This assay recognizes natural and recombinant mouse IL-6 and no significant cross-reactivity or interference was observed against the factors listed below by the manufacturer.
Recombinant mouse: CT-1, gp130, IL-6 sR, IL-11, LIF, OSM
Recombinant human: IL-6, IL-6 sR
Recombinant porcine: IL-6
Recombinant rat IL-6 cross-reacted approximately 0.1% in the assay by the manufacturer.

Eukaryotic cell lines

Policy information about cell lines

Cell line source(s)

MEFs derived from Mff lox/lox mice were developed in Nomura M's lab. Drp1 KO MEFs were described in Ishihara N et al., Nat Cell Biol 11:958-966, 2009. HeLa cells and Mff KO HeLa cells were described in Otera H et al., J Cell Biol 212:531-544, 2016.

Authentication	Mff KO MEFs, Drp1 KO MEFs, Mff KO HeLa cells were subjected to immunoblotting and immunostaining analysis to confirm the loss of protein expression.
Mycoplasma contamination	MEFs and HeLa cell lines have been tested for mycoplasma using a PCR Mycoplasma Detection Set (Takara) and verified as mycoplasma-negative.
Commonly misidentified lines (See ICLAC register)	Cells used in this study are not listed in the database of commonly misidentified cell lines maintained by ICLAC.
